# Supplementary material for: Knowledge, attitude, practice, and fear level of Bangladeshi students toward Covid-19 after a year of the pandemic situation: A web-based cross-sectional study
Source: PLoS One. 2023 Feb 27;18(2):e0282282. doi: 10.1371/journal.pone.0282282 (PMC9970072; doi:10.1371/journal.pone.0282282)
Supplement: S1 File — (DOCX) [file pone.0282282.s001.docx]

|  | | | | | | |
| --- | --- | --- | --- | --- | --- | --- |
| **Questionnaire and Coding information** | | | | | | |
| **Demographic Information** | | | | | | |
| Serial No | | Full variable name/Label | | SPSS variable name | Coding instruction | |
| 01 | | Sex | | Sex | Male=0  Female=1 | |
| 02 | | Age | | Age | 16-30=1 | |
| 03 | | Education | | Education | HSC=0  Honors/Masters=1 | |
| 04 | | Occupation | | Occupation | Student as well as tutor=0  Student only=1 | |
| 05 | | Family | | Family | Nuclear=0  Extended=1 | |
| 06 | | Monthly income | | Income | 20,000 BDT or less=0  21,000-40,000 BDT=1  40,000 BDT=2 | |
| 07 | | Where are you in? (at least for 3 month) | | Location | Village=0  Sub-district=1  District=2  Divisional district=3 | |
| **Knowledge Measuring Scale** | | | | | | |
| 01 | Covid-19 disease is deadly but preventable as well as curable. | | KN01 | | | Right=0  wrong=1  I don’t know=2 |
| 02 | Fever, fatigue, dry cough, and breathing difficulty are main clinical symptoms of covid-19. | | KN02 | | | Right=0  wrong=1  I don’t know=2 |
| 03 | Elderly people having chronic illnesses and other complications are more likely to be seriously affected. | | KN03 | | | Right=0  wrong=1  I don’t know=2 |
| 04 | This disease can be prevented with antibiotic and other medications. | | KN04 | | | Right=0  wrong=1  I don’t know=2 |
| 05 | Person with covid-19 having no symptoms (fever) cannot spread virus to others. | | KN05 | | | Right=0  wrong=1  I don’t know=2 |
| 06 | Corona virus can spread via respiratory droplets (from coughing, sneezing) of infected people. | | KN06 | | | Right=0  wrong=1  I don’t know=2 |
| 07 | Taking measures is not necessary for children and young adults to prevent the infections by corona virus. | | KN07 | | | Right=0  wrong=1  I don’t know=2 |
| 08 | Isolation and treatment of people with covid-19 are effective ways to reduce the spread of the virus. | | KN08 | | | Right=0  wrong=1  I don’t know=2 |
| 09 | People who have contact with someone infected with the COVID-19 virus or came from infected area/country should be immediately isolated for 14 days. | | KN09 | | | Right=0  wrong=1  I don’t know=2 |
| 10 | Covering mouth and nose with bent-elbow or tissue paper or handkerchief when coughing or sneezing can reduce the spread of this virus. | | KN10 | | | Right=0  wrong=1  I don’t know=2 |
| 11 | This disease could be asymptomatic. | | KN11 | | | Right=0  wrong=1  I don’t know=2 |
| 12 | Individuals should avoid going to crowded places such as market and public transportations to prevent the infection. | | KN12 | | | Right=0  wrong=1  I don’t know=2 |
| **Scoring**  Right* = 1  Wrong = 0  I don’t know = 0  [Applicable for 1,2,3,6,9,10,11,13,14,15]  Right =0  Wrong* =1  I don’t know =0  [Applicable for 4,5,7,8,12]  **Knowledge Total Score (KTS)**  **Knowledge Category (KC)**  More Accurate knowledge = 1  Less Accurate Knowledge = 0 | | | | | | |
| **Attitude Measuring Scale** | | | | | | |
| 01 | Do you like to stay at home for certain period (2 weeks) to prevent corona virus spread if government will order so? | | A01 | | | Yes=0  No=1  Not possible=2 |
| 02 | Do you think that social distancing (e.g. stay 1-2 m apart, avoid crowds etc.) can prevent the spread of this virus? | | A02 | | | Yes=0  No=1  May be=2 |
| 03 | Do you agree that we should cancel business/ recreational trips at this time? | | A03 | | | Yes=0  No=1  May be=2 |
| 04 | Do you believe that working from home can help to control covid-19? | | A04 | | | Yes=0  No=1  May be=2 |
| 05 | Do you agree that government should have taken preventive measures when new variants were first reported in Bangladesh? | | A05 | | | Yes=0  No=1  May be=2 |
| 06 | Do you think health education can play an important role in covid-19 prevention? | | A06 | | | Yes=0  No=1  May be=2 |
| 07 | Do you agree that COVID-19 will finally be successfully controlled? | | A07 | | | Yes=0  No=1  May be=2 |
| 08 | Do you have confidence that Bangladesh can win the battle against the COVID-19 virus? | | A08 | | | Yes=0  No=1  May be=2 |
| **Scoring**  For 2,3,4,6,8,9,10 Yes=2, No=0, May be=1  For 1 Yes=2, No=0, Not possible=0  For 5 Yes=0, No =2, Not enough=2  For 7 Yes=0, No =0, May be=2  **Attitude Total Score** **(ATS)**  **Attitude Category (AC)**  More Positive Attitude= 1  Less Positive Attitude= 0 | | | | | | |
| **Practice Assessment Scale** | | | | | | |
| 01 | Do you wash hands with water and soap frequently? | | P01 | | | Yes=0  No=1  Sometimes=2 |
| 02 | Do you always use mask? | | P02 | | | Yes=0  No=1  Sometimes=2 |
| 03 | Do you maintain the rules of using mask? | | P03 | | | Yes=0  No=1  Sometimes=2 |
| 04 | Do you maintain social distance (or home quarantine)? | | P04 | | | Yes=0  No=1  Sometimes=2 |
| 05 | Do you use tissues or hanker chips during coughing/sneezing? | | P05 | | | Yes=0  No=1  Sometimes=2 |
| 06 | Do you eat healthy food or maintain a healthy lifestyle focusing on outbreak? | | P06 | | | Yes=0  No=1  Sometimes=2 |
| 07 | Do you avoid public transports (like bus, train, Plane etc.)? | | P07 | | | Yes=0  No=1  Sometimes=2 |
| 08 | Do you avoid handshaking, hugging when you meet with your friends? | | P08 | | | Yes=0  No=1  Sometimes=2 |
| **Scoring**  For all questions Yes=2, No =0, Sometimes= 1  **Practice Total Score** **(PTC)**  **Practice Category (PC)**  More frequent practice = 1  Less frequent practice score = 0 | | | | | | |
| **Fear Level Test 20** | | | | | | |
| 01 | I am most afraid of coronavirus-19. | | F01 | | | Strongly agree=4  Agree=3  Neutral=2  Disagree=1  Strongly disagree=0 |
| 02 | It makes me uncomfortable to think about coronavirus-19. | | F02 | | | Strongly agree=4  Agree=3  Neutral=2  Disagree=1  Strongly disagree=0 |
| 03 | My hands become clammy when I think about coronavirus-19. | | F03 | | | Strongly agree=4  Agree=3  Neutral=2  Disagree=1  Strongly disagree=0 |
| 04 | I am afraid of losing my life because of coronavirus-19. | | F04 | | | Strongly agree=4  Agree=3  Neutral=2  Disagree=1  Strongly disagree=0 |
| 05 | When watching news and stories about coronavirus-19 on social media, I become nervous or anxious. | | F05 | | | Strongly agree=4  Agree=3  Neutral=2  Disagree=1  Strongly disagree=0 |
| 06 | I cannot sleep because I’m worrying about getting coronavirus-19. | | F06 | | | Strongly agree=4  Agree=3  Neutral=2  Disagree=1  Strongly disagree=0 |
| 07 | My heart races or palpitates when I think about getting coronavirus-19. | | F07 | | | Strongly agree=4  Agree=3  Neutral=2  Disagree=1  Strongly disagree=0 |
| 08 | Are you maintaining all safety rules for covid-19? | | C0 | | | Yes=0  No=1 |
| **Scoring**  *Strongly agree=4  *Agree=3  Neutral=2  Disagree=1  Strongly disagree=0  **Fear Total Score (FTS)**  **Fear Category 1 (FC 1)**  Less fear = 0  Moderate fear = 1  High fear = 2  **Fear Category 2 (FC 2)**  Moderate to High fear = 0  Very little fear = 1 | | | | | | |
| **Mention causes exactly why you can’t maintain all safety rules.** | | | | | | |
| 00 | Do you maintain public health hygiene properly? | | C0 | | | Yes=0  No=1 |
| 01 | Beliefs that I will not be infected by corona virus. | | C01 | | | Yes = 0  No = 1  No response = 99 |
| 02 | Things related with corona seems to me as media-created rumors. | | C02 | | | Yes = 0  No = 1  No response = 99 |
| 03 | No one having been identified as corona virus positive yet from my relatives or relatives of relatives or neighbors makes maintaining safety rules less important to me. | | C03 | | | Yes = 0  No = 1  No response = 99 |
| 04 | I am less panicked about corona virus. | | C04 | | | Yes = 0  No = 1  No response = 99 |
| 05 | I have no clear conception about safety rules for covid-19. | | C05 | | | Yes = 0  No = 1  No response = 99 |
| 06 | It is not always possible for me to wash hands coming from outsides. | | C06 | | | Yes = 0  No = 1  No response = 99 |
| 07 | Very few times in a day hands are washed up to elbow for (20-30) min as recommended by WHO. | | C07 | | | Yes = 0  No = 1  No response = 99 |
| 08 | I do not use hand sanitizer when staying outside. | | C08 | | | Yes = 0  No = 1  No response = 99 |
| 09 | Trying but I can’t give up the bad habit of touching eyes, face, nose in frequent. | | C09 | | | Yes = 0  No = 1  No response = 99 |
| 10 | I am to go market often for daily groceries or other things where maintaining social distance is not possible. | | C10 | | | Yes = 0  No = 1  No response = 99 |
| 11 | I am to go out for many reasons and use public transports where maintaining social distance is not possible. | | C11 | | | Yes = 0  No = 1  No response = 99 |
| 12 | I am social butterfly and to hang out with friends very often while social distance is not maintained. | | C12 | | | Yes = 0  No = 1  No response = 99 |
| 13 | I don’t maintain social distance thinking the benefit of maintaining social distance when people around me don’t maintain social distance. | | C13 | | | Yes = 0  No = 1  No response = 99 |
| 14 | I don’t use medical mask because of having no confidence on normal medical mask (5 BDT) as well as no money for buying mask frequently. | | C14 | | | Yes = 0  No = 1  No response = 99 |
| 15 | When wearing mask, I feels very uneasy and it’s very hard to stay with. | | C15 | | | Yes = 0  No = 1  No response = 99 |
| 16 | I don’t use mask because it looks me ugly. | | C16 | | | Yes = 0  No = 1  No response = 99 |
| 17 | I don’t know any reason. | | C17 | | | Yes = 0  No = 1  No response = 99 |
