# Supplementary material for: Knowledge, attitude, practice, and fear level of Bangladeshi students toward Covid-19 after a year of the pandemic situation: A web-based cross-sectional study
Source: PLoS One. 2023 Feb 27;18(2):e0282282. doi: 10.1371/journal.pone.0282282 (PMC9970072; doi:10.1371/journal.pone.0282282)
Supplement: Attachment — Submitted filename: PONE-D-21-23855_R3_reviewer1_ comments.pdf [file pone.0282282.s009.pdf]

PLOS ONE

Knowledge, attitude, practice, and fear level of Bangladeshi students towards Covid-19 after a year of the pandemic situation: a web-based cross-sectional study.

--Manuscript Draft--

**Manuscript Number:**	PONE-D-21-23855R3
**Article Type:**	Research Article
**Full Title:**	Knowledge, attitude, practice, and fear level of Bangladeshi students towards Covid-19 after a year of the pandemic situation: a web-based cross-sectional study.
**Short Title:**	Knowledge, attitude, practice, and fear level of Bangladeshi Students towards Covid-19.
**Corresponding Author:**	Tahsin Ahmed Rupok<br>University of Rajshahi<br>BANGLADESH
**Keywords:**	knowledge; Attitude; practice; fear; COVID-19; Bangladesh
**Abstract:**	In the earlier phase of the pandemic situation, the Government of Bangladesh (GoB) badly suffered to adhere their people to preventive measures probably due to less knowledge and attitude towards Covid-19. To tackle the second wave of coronavirus, GoB again enforced an array of preventive measures, but still encountering the same problem after a year of the pandemic situation. As an attempt to find out the reasons behind this, our study aimed to assess the present knowledge, attitude, practice, and fear level of the students. A cross-sectional study was designed and conducted from 15 th to 25 th April 2021. A total of 382 participants met all the inclusion criteria and were considered for performing all the statistical analyses (Descriptive statistics, Mann-Whitney U test, Kruskal-Wallis H test, Multiple logistic regression, Spearman rank-order correlation). All the participants were students aged 16 to 30. 84.8%, 66%, 55%, and 22.3% participants had more accurate knowledge, more positive attitude, more frequent practice, and moderate to high fear towards Covid-19, respectively. Knowledge, attitude, practice, and fear were interrelated directly or indirectly. It was found knowledgeable participants were more likely to have more positive attitude (AOR = 2.34, 95% CI = 1.23-4.47, P < 0.01) and very little fear (AOR = 2.17, 95% CI = 1.10-4.26, P < 0.05). More positive attitude was found as a good predictor of more frequent practice (AOR = 4.00, 95% CI = 2.44-6.56, P < 0.001), and very less fear had negative impact on both attitude (AOR = 0.44, 95% CI = 0.23-0.84, P < 0.01) and practice (AOR = 0.47, 95% CI = 0.26-0.84, P < 0.01). The findings reflect that students had appreciable knowledge and very little fear, but disappointedly had average attitude and practice towards Covid-19 preventive measures (CPMs). In addition, students had lack of confidence on Bangladesh would win the battle against Covid-19. Thus, our findings make a recommendation that policymakers should be more focused to scale up students' confidence and attitude towards CPMs by developing and implementing well-conceived plan of actions besides insisting them to practice CPMs.
**Order of Authors:**	Tahsin Ahmed Rupok
	Sunandan Dey
	Rashni Agarwala
	Md. Nurnobi Islam
	Bayezid Bostami
**Response to Reviewers:**	Dear editor,<br>Here I am writing our responses to each points raised by the reviewers.<br><br>Responses to the queries of Reviewer #1:<br>Thanks for your valuable comments. According to your suggestion, all the authors critically revised the manuscript again. Besides, we requested another expert (name mentioned on the Acknowledgment section) to revise our manuscript. Hopefully, his contribution would add significant value to this manuscript. (All the changes have been marked on the "Revised Manuscript with Track Changes")

Powered by Editorial Manager® and ProduXion Manager® from Aries Systems Corporation

	Responses to the queries of Reviewer #2:<br>Thanks for your valuable suggestion. I provided the approval letter (scanned copy of the original letter) as a supplementary file for strengthening the credibility of ethical issue of the study.
**Additional Information:**	
**Question**	**Response**
**Financial Disclosure**<br><br>Enter a financial disclosure statement that describes the sources of funding for the work included in this submission. Review the submission guidelines for detailed requirements. View published research articles from *PLOS ONE* for specific examples.<br><br>This statement is required for submission and **will appear in the published article** if the submission is accepted. Please make sure it is accurate.<br><br>**Unfunded studies**<br>Enter: *The author(s) received no specific funding for this work.*<br><br>**Funded studies**<br>Enter a statement with the following details:<br>• Initials of the authors who received each award<br>• Grant numbers awarded to each author<br>• The full name of each funder<br>• URL of each funder website<br>• Did the sponsors or funders play any role in the study design, data collection and analysis, decision to publish, or preparation of the manuscript?<br>• **NO** - Include this sentence at the end of your statement: *The funders had no role in study design, data collection and analysis, decision to publish, or preparation of the manuscript.*<br>• **YES** - Specify the role(s) played.<br><br>* typeset	The authors received no specific funding for this work.
**Competing Interests**<br><br>Use the instructions below to enter a competing interest statement for this submission. On behalf of all authors, disclose any competing interests that could be perceived to bias this	The authors have declared that no competing interests exist.

work—acknowledging all financial support and any other relevant financial or non-financial competing interests.<br><br>This statement is required for submission and **will appear in the published article** if the submission is accepted. Please make sure it is accurate and that any funding sources listed in your Funding Information later in the submission form are also declared in your Financial Disclosure statement.<br><br>View published research articles from *PLOS ONE* for specific examples.<br><br>**NO authors have competing interests**<br><br>Enter: *The authors have declared that no competing interests exist.*<br><br>**Authors with competing interests**<br><br>Enter competing interest details beginning with this statement:<br><br>*I have read the journal's policy and the authors of this manuscript have the following competing interests: [insert competing interests here]*<br><br>* typeset	
**Ethics Statement**<br><br>Enter an ethics statement for this submission. This statement is required if the study involved:<br><br>• Human participants<br>• Human specimens or tissue<br>• Vertebrate animals or cephalopods<br>• Vertebrate embryos or tissues<br>• Field research<br><br>Write "N/A" if the submission does not require an ethics statement.	This study was conducted according to the principles expressed in the declaration of Helsinki. Besides, this study received ethical approval from Institute of Biological Science, University of Rajshahi, Bangladesh (ERC Memo no: 91/320IAMEBBC/IBSc). Anonymity and confidentiality were strictly maintained. All respondents gave full informed consent to participate and consent for their data to be used in the publication. Participants aged under 18 years old were requested to provide their guardians' Email id or Messenger id or Whatsapp id or Phone number so that authors or volunteers can contact their guardians to inform them about the objectives of the survey, and take consents from them regarding their children's participation. This study included only those participants aged under 18 years old who made their guardians contact with authors or volunteers.

Powered by Editorial Manager® and ProduXion Manager® from Aries Systems Corporation

General guidance is provided below. Consult the submission guidelines for detailed instructions. **Make sure that all information entered here is included in the Methods section of the manuscript.**<br><br>**Format for specific study types**<br><br>**Human Subject Research (involving human participants and/or tissue)**<br>• Give the name of the institutional review board or ethics committee that approved the study<br>• Include the approval number and/or a statement indicating approval of this research<br>• Indicate the form of consent obtained (written/oral) or the reason that consent was not obtained (e.g. the data were analyzed anonymously)<br><br>**Animal Research (involving vertebrate animals, embryos or tissues)**<br>• Provide the name of the Institutional Animal Care and Use Committee (IACUC) or other relevant ethics board that reviewed the study protocol, and indicate whether they approved this research or granted a formal waiver of ethical approval<br>• Include an approval number if one was obtained<br>• If the study involved *non-human primates*, add *additional details* about animal welfare and steps taken to ameliorate suffering<br>• If anesthesia, euthanasia, or any kind of animal sacrifice is part of the study, include briefly which substances and/or methods were applied<br><br>**Field Research**<br><br>Include the following details if this study involves the collection of plant, animal, or other materials from a natural setting:<br>• Field permit number<br>• Name of the institution or relevant body that granted permission	
**Data Availability**<br><br>Authors are required to make all data underlying the findings described fully	Yes - all data are fully available without restriction

available, without restriction, and from the time of publication. PLOS allows rare exceptions to address legal and ethical concerns. See the PLOS Data Policy and FAQ for detailed information.<br><br>A Data Availability Statement describing where the data can be found is required at submission. Your answers to this question constitute the Data Availability Statement and **will be published in the article**, if accepted.<br><br>**Important:** Stating 'data available on request from the author' is not sufficient. If your data are only available upon request, select 'No' for the first question and explain your exceptional situation in the text box.<br><br>Do the authors confirm that all data underlying the findings described in their manuscript are fully available without restriction?	
**Describe where the data may be found in full sentences. If you are copying our sample text, replace any instances of XXX with the appropriate details.**<br><br>• If the data are **held or will be held in a public repository**, include URLs, accession numbers or DOIs. If this information will only be available after acceptance, indicate this by ticking the box below. For example: *All XXX files are available from the XXX database (accession number(s) XXX, XXX.*).<br>• If the data are all contained **within the manuscript and/or Supporting Information files**, enter the following: *All relevant data are within the manuscript and its Supporting Information files.*<br>• If neither of these applies but you are able to provide **details of access elsewhere**, with or without limitations, please do so. For example:<br><br>*Data cannot be shared publicly because of [XXX]. Data are available from the XXX Institutional Data Access / Ethics*	All relevant data are within the manuscript and its supporting information files.

Committee (contact via XXX) for researchers who meet the criteria for access to confidential data.<br><br>*The data underlying the results presented in the study are available from (include the name of the third party and contact information or URL).*<br>• This text is appropriate if the data are owned by a third party and authors do not have permission to share the data.<br><br>* typeset	
Additional data availability information:	

Powered by Editorial Manager® and ProduXion Manager® from Aries Systems Corporation

Manuscript Click here to access/download;Manuscript;Manuscript.docx

Knowledge, attitude, practice, and fear level of Bangladeshi students towards Covid-19 after a year of the pandemic situation: a web-based cross-sectional study.

Short title: Knowledge, attitude, practice, and fear level of Bangladeshi Students towards Covid-19.

Authors and affiliations:

1. Tahsin Ahmed Rupok**, Email: tahsinahmedrupokpharmru9176@gmail.com

Department of Pharmacy, University of Rajshahi, Rajshahi-6205, Bangladesh.

2. Sunandan Dey, Email: sunandandeypharmacy@gmail.com

Department of Pharmacy, University of Rajshahi, Rajshahi-6205, Bangladesh.

3. Rashni Agarwala, Email: rashniagarwala@gmail.com

Department of Pharmacy, Islamic University, Kushtia-7003, Bangladesh.

4. Md. Nurnobi Islam, Email: nurnobi6329@gmail.com

Department of Chemistry, Shahjalal University of Science & Technology, Sylhet-3114, Bangladesh.

5. Bayezid Bostami, Email: bayezidpharm.ru@gmail.com

Department of Pharmacy, University of Rajshahi, Rajshahi-6205, Bangladesh

**Corresponding author: tahsinahmedrupokpharmru9176@gmail.com

Abstract

In the earlier phase of the pandemic situation, the Government of Bangladesh (GoB) badly suffered to adhere their people to preventive measures probably due to less knowledge and attitude towards Covid-19. To tackle the second wave of coronavirus, GoB again enforced an array of preventive measures, but still encountering the same problem after a year of the pandemic situation. As an attempt to find out the reasons behind this, our study aimed to assess the present knowledge, attitude, practice, and fear level of the students. A cross-sectional study was designed and conducted from 15th to 25th April 2021. A total of 382 participants met all the inclusion criteria and were considered for performing all the statistical analyses (Descriptive statistics, Mann-Whitney U test, Kruskal-Wallis H test, Multiple logistic regression, Spearman rank-order correlation). All the participants were students aged 16 to 30. 84.8%, 66%, 55%, and 22.3% participants had more accurate knowledge, more positive attitude, more frequent practice, and moderate to high fear towards Covid-19, respectively. Knowledge, attitude, practice, and fear were interrelated directly or indirectly. It was found knowledgeable participants were more likely to have more positive attitude (AOR = 2.34, 95% CI = 1.23-4.47, $P < 0.01$) and very little fear (AOR = 2.17, 95% CI = 1.10-4.26, $P < 0.05$). More positive attitude was found as a good predictor of more frequent practice (AOR = 4.00, 95% CI = 2.44-6.56, $P < 0.001$), and very less fear had negative impact on both attitude

(AOR = 0.44, 95% CI = 0.23-0.84, $P < 0.01$) and practice (AOR = 0.47, 95% CI = 0.26-0.84, $P < 0.01$). The findings reflect that students had appreciable knowledge and very little fear, but disappointedly had average attitude and practice towards Covid-19 preventive measures ~~(CPMs)~~. In addition, students had lack of confidence on Bangladesh would win the battle against Covid-19. Thus, our findings make a recommendation that policymakers should be more focused to scale up students' confidence and attitude towards CPMs by developing and implementing well-conceived plan of actions besides insisting them to practice CPMs.

Keywords: Knowledge; Attitude; Practice; Fear; Covid-19; Bangladesh.

Introduction

In Bangladesh, both infection and death rates have started to burgeon again from the second week of March 2021 with a small dip in late April, which has been further instigated due to the entrance of highly transmissible Delta variant of coronavirus in May 2021(1). As of June 29, 2021, 7,666 new cases were detected with a positivity rate of 23.97%, and 112 people lost their life (2). Delta variant, first detected in India in late 2020, is highly contagious and deadly (3). If this Delta variant starts to escalate vehemently in Bangladesh as it did in India and Nepal, people stay lackadaisical in maintaining public health hygiene, and governments continue to

develop an ill-conceived plan of action and fail to ensure proper implementation of preventive measures, then, the number of cases detected per day could rise to an uncontrollable digit, which can throw the situation into turmoil (4). So, the government should immediately take the necessary steps to prevent any grim situation. It is worth mentioning that prevention is the best and comparatively less costly method to the most effective protection against the virus. Only adequate preventive measures can keep the infection rate under control, which will truncate both the number of patients and death tallies. As a preventive measure, the government decided to go for countrywide strict lockdown again from 1st July 2021 but it is worth remembering that lockdowns are only effective if they are accompanied by other preventive measures (5,6). However, the GoB badly suffered to adhere their population to preventive measures in the earlier phase of the pandemic situation probably due to people's less knowledge and attitude to embrace the measures taken by the governments. A review of some previous studies conducted in Bangladesh reflected the truth that in last year (2020) Bangladeshi people were comparatively less knowledgeable, had a less positive attitude toward Covid-19, and as a consequence had less frequent practice, which was consistent with a study of Pakistan but inconsistent with China and Saudi Arabia (7–14). Indeed, there are also exceptions such as one article from Bangladesh (9) showed people (78.9%) had a comparatively more positive attitude towards Covid-19.

Moreover, another article from Bangladesh (8) reported that people had more frequent practice (94.3%) along with more positive attitude (93.0%) towards CPMs (Fig 1).

However, one thing to remember, adhering people to preventive measures is not straightforward, takes intensive efforts over a length of time as well as depends on what extent people can perceive the importance of those measures. So, proper implementation of these preventive measures warrants adequate well-planned initiatives to scale up people's knowledge and awareness towards Covid-19 and its preventive measures, which will ultimately alter their behavior as well as attitude positively towards Covid-19 preventive practice. Several studies published last year (2020) outlined that adequate knowledge regarding Covid-19 is a potential predictor of attitude towards its preventive practice (13,15). To speak the truth, it is indefinite when the coronavirus will go away from the world. Therefore, people must be resilient to this new situation, learn all fundamental information regarding Covid-19, and change their attitude positively towards practicing all safety guidelines stipulated by their government.

Fig 1 Diagram reflecting different countries and their peoples' knowledge, attitude, and practice level towards covid-19. ***[Bangladesh 1*** (7)***, Bangladesh 2*** (11)***, Bangladesh 3*** (13)***, Bangladesh 4*** (9)***, Bangladesh 5 (8), Pakistan (12), Saudi Arabia*** (14)***, China*** (10)***].***

However, since the emergence of the pandemic situation, the GoB have been confronting troubles to aware and adhere their people to CPMs. After a year of pandemic situation, the situation seems to remain stagnant and the GoB is still facing the same problem. Therefore, it is needed to explore the present KAP level of Bangladeshi people. Anyway, this study aimed to bring up the present KAP level of Bangladeshi students and how well-prepared they are to confront any future onslaught of Covid-19. So, the objectives of this present study were-

-To explore the present knowledge, attitude, practice, and fear level of the Bangladeshi students and their associated potential factors.

-To find out whether knowledge, attitude, practice, and fear are related to each other.

-To find out the most probable reasons why students are digressed from maintaining CPMs enforced by the governments.

Materials and Methods

Participants and Data recruitment procedure

This web-based cross-sectional survey was conducted on Bangladeshi students using online facilities amid the second wave of coronavirus in Bangladesh, continued for 10 days from 15th to 25th April in 2021. A structured questionnaire was prepared in a Google Form and a link was generated, shared with all authors and other volunteers who were instructed properly as to how well they can use this form to recruit data

with adequate consent from the participants and from the guardians of the participants aged under 18 years old. A face-to-face community-based survey was not possible as the GoB implemented their second strict lockdown from 14th April being one day ahead of our data collection initiated (16), all data were therefore assembled through online approaches using Whatsapp, Messenger, Email, Facebook. This survey included subjects aged 16 years old or above, being Bangladeshi residents, having internet access and ability to communicate, having no intellectual disability. Subjects living in foreign countries, having no or less interest to participate in were excluded from the survey. A total of 382 participants successfully submitted their data and all completed the questionnaire adequately as they were recommended.

Survey Instrument

The questionnaire was prepared after a thorough review of some literature published last year (2020), which had following six sections.

Preface

This section had a brief delineation of the objectives of the survey and some instructions on how to complete the questionnaire properly. It also included a question asked to take consent of the respondents.

Socio-demographic Measures

This section embodied 7 questions asked to gather socio-demographic information of a participant. Socio-demographic information included gender, age, education, occupation, location, family type, family income which was categorized as follows (<20,000 BDT), (20,000-40,000 BDT), (>40,000 BDT).

Knowledge Scale

This section had 12 questions where each question had three options “Right”, “Wrong”, and “Not sure”. During scoring, responses of the questions with “Wrong” as a correct answer (Table 2) were first recoded from “Wrong” to “Right”. Then, the correct answer (Right) was coded as 1 and the incorrect answer (Wrong/Not sure) was coded as 0. The total score ranged from 0 to 12 where those who obtained 83% score or greater (≥ 10) were considered to have more accurate knowledge otherwise considered having less accurate knowledge. Instead of considering 80% score for more accurate knowledge based on Bloom’s cut off point, 83% score was considered to avoid fractional digits as all the knowledge scores were integer numbers.

Attitude Scale

This section had 8 questions where each question had three options “Yes”, “No”, and “Maybe” except one question having “Not possible” instead of “Maybe” ~~in three options~~ (Table 2). During scoring, four kinds of options were coded as follows, 0

(No/ Not possible for work), 1 (Maybe), and 2 (Yes). The total score ranged from 0 to 16. Those who had an 81% score or greater (≥ 13) were considered to have a positive attitude otherwise considered to have a less positive attitude. Instead of considering 80% score for positive attitude based on Bloom's cut off point, 81% score was considered to avoid fractional digits as all the attitude scores were integer numbers.

Practice Scale

This section also had 8 questions where each question had three options "Yes", "No", and "Sometimes". During scoring, three options were coded as follows, 0 (No), 1 (Sometimes), and 2 (Yes). The total score ranged from 0 to 16. Those who had an 81% score or greater (≥ 13) were considered to have more frequent practice otherwise considered having less frequent practice. Instead of considering 80% score for more frequent practice based on Bloom's cut off point, 81% score was considered to avoid fractional digits as all the practice scores were integer numbers.

Fear Scale

This section included a fear measuring tool which was reported as a valid and reliable instrument to assess Covid-19 related fear among respondents (17). This tool had 7 items where each item had five Likert options which were coded as follows, 0 (Strongly disagree), 1 (Disagree), 2 (Neutral), 3 (Agree), 4 (Strongly Agree). The

total score ranged from 0 to 28. Fear scores greater or equal to 21 were cut-points for high fear. Scores between 20-14 were cut points for moderate fear and scores equal to or less than 13 were cut points for less fear.

This section also had two extra questions. The first question having binary options "Yes", "No" asked if or not they are maintaining preventative measures properly stipulated by the governments. The second question was asked to those who answered "No" to account for their reasons why they are aberrant than those who answered "Yes". There were 17 possible responses and multiple responses could be selected.

Reliability & Validity

Reliability tests of the instrument were performed using field data to determine the validity and reliability of those scales mentioned above. Cronbach's alpha for knowledge, attitude, practice, and fear scale were 0.47, 0.61, 0.81, and 0.83. Due to a shortage of time, we could not conduct a pilot study.

Ethical considerations

This study was conducted according to the principles expressed in the declaration of Helsinki. Besides, this study received ethical approval from Institute of Biological Science, University of Rajshahi, Bangladesh (ERC Memo no: 91/320IAMEBBC/IBSc). Anonymity and confidentiality were strictly maintained.

All respondents gave full informed consent to participate and consent for their data to be used in the publication. Participants aged under 18 years old were requested to provide their guardians' Email id or Messenger id or Whatsapp id or Phone number so that authors or volunteers can contact their guardians to inform them about the objectives of the survey, and take consents from them regarding their children's participation. This study included only those participants aged under 18 years old who made their guardians contact with authors or volunteers.

Data Management and Analysis

All submitted data were automatically stored in a dynamic Microsoft excel sheet which was made available offline after completion of data collection for primary data processing including data duplication-checking, data cleaning, data coding, etc. In this stage, three data fallen out of inclusion criteria of age (below 16 years old), and two data got duplicated were discard from the preliminary dataset. After accomplishment of data cleaning and coding, the final dataset having data of 382 respondents was entered into Statistical Package for the Social Science (SPSS) version 26.0 for further data processing and doing all the statistical analysis which included descriptive statistics (to determine percentage, mean, standard deviation), Mann-Whitney U test (to determine difference between two groups under the same

variable having two groups), Kruskal Wallis H test (to determine difference among the groups under same variable having more than two groups), Spearman rank-order correlation (if variables are ordinal), Multiple logistic regression (to determine predictors for accurate knowledge, positive attitude, frequent practice, very little fear) including gender, education, occupation, family type, family income, location, knowledge, attitude, practice, fear as independent variables. Non-parametric Mann-Whitney U test and Kruskal Wallis H test, and Spearman rank-order correlation were performed as the data under continuous dependent variables were not normally distributed. To check that, besides visual inspection of histograms, Shapiro-Wilk and Kolmogorov-Smirnov tests were conducted. Both tests were statistically significant at 0.01 for those continuous dependent variables and histograms of them looked left-skewed, which confirmed well that data under these variables were not normally distributed. For all statistical analyses noted above, the statistical significance level was set to 0.05 (Alpha value).

Results

Demographic characteristics of the sample

This study considered a total of 382 participants for final statistical analysis. Among them majority were male (74.6%), studying in BSc and MSc (71.8%). All the participants were students while 39.8% were self-employed. About 76.7% and

58.1% of the participants were from nuclear and lower income family (<20,000 BDT) respectively. 37.2% of the participants informed their location village. This study inclined towards the adult population as all participants had ages of 16 to 30 years old. (Table 1) tabulated detailed information about the socio-demographic characteristics of the respondents.

Table 1

Socio-demographic characteristics of the respondents.

Characteristics		Frequency	Percentage (%)
Gender	Male	285	74.6
	Female	97	25.4
Education	HSC	104	27.2
	BSc/MSc/Above	278	71.8
Occupation	Student and self-employed	152	39.8
	Student only	230	60.2
Family type	Nuclear	293	76.7
	Extended	89	23.3
Income	<20,000 BDT	222	58.1
	20,000-40,000 BDT	126	33.0
	>40,000 BDT	34	8.9
Location	Village	142	37.2
	Sub-district town	39	10.2
	District town	97	25.4
	Divisional district town	104	27.2

HSC- Higher Secondary School Certificate, BSc- Bachelor of Science, MSc- Masters of Science

Knowledge and its factors

Participants' Overall correct answer rate was 89.6% [Mean Score (MS) ± Standard Deviation (SD) = 10.76±1.20 out of 12] indicating participants had appreciable knowledge on Covid-19. 84.8% of the participants had more accurate knowledge

while only 15.2% had less accurate knowledge. The distribution of responses for each question of the knowledge scale was presented in (Table 2). Mann-Whitney and Kruskal-Wallis tests showed that knowledge scores do not vary with any socio-demographic variables included in our study, indicating all the groups of a variable had quite similar knowledge on Covid-19 disease (Table 4). Multiple logistic regression analysis identified location, attitude, and fear as significant factors that can predict if an individual would have more accurate knowledge on Covid-19 ~~rather than having less accurate knowledge~~ (Table 5).

Attitude and its factors

On average, participants had an 82% score on the attitude scale (MS ± SD = 13.14 ± 2.21 out of 16). Approximately 66% of the participants had ~~a~~ more positive attitude. Only 35.3% of the participants had confidence that Bangladesh can win the battle against Covid-19 and only 44.2% agreed that Covid-19 will finally be controlled. The distribution of responses for each question of the attitude scale was presented in (Table 2). Mann-Whitney and Kruskal-Wallis tests showed attitude scores vary with occupation (P= 0.047) and social status (P= 0.05). It was found that the participants who are ~~student and~~ self-employed, and came from a lower income family (<20,000 BDT) had significantly less positive attitude than their counterparts. (Table 4). Multiple logistic regression analysis identified income, location, knowledge, practice, fear, as significant factors that can predict if an individual would have a

more positive attitude rather than a less positive attitude. It was found that the participants who came of a lower income family (<20,000 BDT) were 67% less likely to have more positive attitude than those who came of a higher income family (> 40,000 BDT) (AOR = 0.33, 95% CI = 0.11-0.94, P < 0.05) and the participants came from divisional district town were 50% less likely to have more positive attitude than those came from village (AOR = 0.50, 95% CI = 0.27-0.93, P < 0.05) (Table 5).

Practice and its factors

On average, participants had a 74.2% score on the practice scale (MS ± SD = 11.87 ± 3.80 out of 16). Approximately 55% of the participants had more frequent practice. The distribution of responses for each question of the practice scale was presented in (Table 2). Mann-Whitney and Kruskal-Wallis tests uncovered that Practice scores vary with gender (P < 0.001), income (P < 0.001), and location (P < 0.01). It was found that females had significantly higher practice score than males (P < 0.001); Participants came of both higher income family (P < 0.01) and middle income family (P < 0.01) had significantly higher practice score than those came of a lower income family; Participants currently living in any district town and sub-district town (P ≤ 0.01) had significantly higher score on practice scale than those living in a village (Table 4). Multiple logistic regression analysis identified gender, location, attitude, fear, as significant factors that can predict if an individual would have more frequent

practice rather than less frequent practice. It was found that males were less likely to have more frequent practice than females by 49% (AOR = 0.51, 95% CI = 0.29-0.89, P $\leq$ 0.05). The participants living in any district town or any divisional district town were 2 times more likely to have more frequent practice than those living in a village (AOR = 2.01, 95% CI = 1.09-3.65, P $\leq$ 0.05);(AOR = 2.04, 95% CI = 1.23-4.47, P $\leq$ 0.01) (Table 5).

Table 2

Participants' knowledge, attitude, and practice towards covid-19 (N = 382) (Distribution of Responses)

Knowledge Questions (Correct Answer)	Right n (%)	Wrong n (%)	Not Sure n (%)
Covid-19 disease is deadly but preventable as well as curable. (Right)	363 (95)	7 (1.8)	12 (3.1)
Fever, fatigue, dry cough, and breathing difficulty are the main clinical symptoms of Covid-19. (Right)	357 (93.5)	11 (2.9)	14 (3.7)
Elderly people having chronic illnesses and other complications are more likely to be seriously affected. (Right)	370 (96.5)	2 (0.5)	10 (2.5)
This disease can be prevented with antibiotics and other medications. (Wrong)	89 (23.3)	189 (49.5)	104 (27.2)
A person with Covid-19 having no symptoms (fever) cannot spread the virus to others. (Wrong)	30 (7.9)	314 (82.2)	38 (9.9)
Coronavirus can spread via respiratory droplets (from coughing, sneezing) of infected people. (Right)	370 (96.9)	8 (2.1)	4 (1.0)
Taking measures is not necessary for children and young adults to prevent infections by the coronavirus. (Wrong)	7 (1.8)	360 (94.2)	15 (3.9)
Isolation and treatment of people with Covid-19 are effective ways to reduce the spread of the virus. (Right)	354 (92.7)	14 (3.7)	14 (3.7)
People who have contact with someone infected with the coronavirus or came from an infected area/country should be immediately isolated for 14 days. (Right)	374 (97.9)	4 (1.0)	4 (1.0)
Covering the mouth and nose with a bent elbow or tissue paper or handkerchief when coughing or sneezing can reduce the spread of this virus. (Right)	357 (93.5)	16 (4.2)	9 (2.4)
This disease could be asymptomatic. (Right)	328 (85.9)	23 (6.0)	31 (8.1)
Individuals should avoid going to crowded places such as markets and public transportations to prevent infection. (Right)	374 (97.9)	6 (1.6)	2 (0.5)
**Table 02 (Continued)**			
Attitude Questions	Yes n (%)	No n (%)	Maybe/Not Possible for work n (%)
Do you like to stay at home for a certain period (2 weeks) to prevent coronavirus spread if the government will order so? **	330 (86.4)	24 (6.3)	28 (7.3)

Do you think that social distancing (e.g. stay 1-2 m apart, avoid crowds, etc.) can prevent the spread of this virus?	320 (83.8)	14 (3.7)	48 (12.6)
Do you agree that we should cancel business/ recreational trips at this time?	249 (65.2)	84 (22.0)	49 (12.8)
Do you believe that working from home can help to control Covid-19?	284 (74.3)	40 (10.5)	58 (15.2)
Do you agree that government should have taken preventive measures when new variants were first reported in Bangladesh?	364 (95.3)	3 (0.8)	15 (3.9)
Do you think health education can play an important role in Covid-19 prevention?	357 (93.5)	7 (1.8)	18 (4.7)
Do you agree that Covid-19 will finally be successfully controlled?	169 (44.2)	28 (7.3)	185 (48.4)
Do you have confidence that Bangladesh can win the battle against the Covid-19 disease?	135 (35.3)	52 (13.6)	195 (51.0)
**Table 02 (Continued)**			
Practice Questions	Yes n (%)	No n (%)	Sometimes n (%)
Do you wash hands with water and soap frequently?	241 (63.1)	30 (7.9)	111 (29.1)
Do you always use a mask?	305 (79.8)	20 (5.2)	57 (14.9)
Do you maintain the rules of using a mask?	304 (79.6)	23 (6.0)	55 (14.4)
Do you maintain social distance (or home quarantine)?	229 (59.9)	66 (17.3)	87 (22.8)
Do you use tissues or hanker chips during coughing/sneezing?	228 (59.7)	89 (23.3)	65 (17.0)
Do you eat healthy food or maintain a healthy lifestyle focusing on outbreaks?	255 (66.8)	40 (10.5)	87 (22.8)
Do you avoid public transports (like buses, trains, planes, etc.)?	169 (44.2)	123 (32.2)	90 (23.6)
Do you avoid handshaking, hugging when you meet with your friends?	223 (58.4)	83 (21.7)	76 (19.9)

** This question had “Not possible for work” instead of “Maybe” in its three options. n- Number of respondents, %- Percentage of respondents, N- Total number of respondents

Fear and its factors

On average, participants had a 35.8% fear score (MS ± SD = 10.03 ± 4.77 out of 28). 77.7% (297) participants had less fear, 20.2% (77) had moderate fear, and only 2.1% (8) participants had high fear. ~~And~~ 77.7% (297) participants had very less fear (less than 50% fear score) while only 22.3% (85) participants had high to moderate fear (equal or more than 50% fear score). The distribution of responses of all fear-related questions was presented in (Table 3). Mann-Whitney and Kruskal-Wallis tests unfolded that fear score among participants varies with only gender ($P < 0.001$). It

was reported that females had a significantly high fear score than males (Table 4). Multiple logistic regression analysis detected gender, knowledge, attitude, practice, as significant factors ~~that can predict whether an individual would have~~ very little fear ~~rather than high to moderate fear.~~ It was found that male participants were 89% more likely to have very less fear than female participants (AOR = 1.89, 95% CI = 1.05-3.40, $P < 0.05$) (Table 5).

Table 3

Fear related to covid-19 among respondents (N= 382) (Distribution of responses).

Fear Questions	Strongly Disagree n (%)	Disagree n (%)	Neutral n (%)	Agree n (%)	Strongly Agree n (%)
I am most afraid of coronavirus-19.	43 (11.3)	96 (25.1)	124 (32.5)	90 (23.6)	29 (7.6)
It makes me uncomfortable to think about coronavirus-19.	35 (9.2)	118 (30.9)	93 (24.3)	109 (28.5)	27 (7.1)
My hands become clammy when I think about coronavirus-19.	119 (31.2)	216 (56.5)	29 (7.6)	15 (3.9)	3 (0.8)
I am afraid of losing my life because of coronavirus-19.	47 (12.3)	134 (35.1)	100 (26.2)	81 (21.2)	20 (5.2)
When watching news and stories about coronavirus-19 on social media, I become nervous or anxious.	45 (11.8)	117 (30.6)	103 (27.0)	86 (22.5)	31 (8.1)
I cannot sleep because I'm worried about getting coronavirus-19.	132 (34.6)	204 (53.4)	32 (8.4)	13 (3.4)	1 (0.3)
My heart races or palpitates when I think about getting coronavirus-19.	127 (33.2)	180 (47.1)	49 (12.8)	24 (6.3)	2 (0.5)

n- Number of respondents, %- Percentage of respondents, N- Total number of respondents

Relationship among knowledge, attitude, practice, and fear

Spearman rank-order correlation tests explored that knowledge score is significantly correlated with attitude score ($P < 0.01$) although it was insignificantly correlated with other scores (practice and fear score). However, attitude score was positively correlated with both practice score ($P < 0.001$) and fear score ($P < 0.001$). Along with attitude score, practice score and fear score was positively correlated with each

other (P < 0.01) (Table 6). Multiple logistic regression analysis uncovered that the participants who had more accurate knowledge were 2.34 times more likely to have a more positive attitude (AOR = 2.34, 95% CI = 1.23-4.47, P < 0.01) and 2.17 times more likely to have very little fear (AOR = 2.17, 95% CI = 1.10-4.26, P < 0.05) than those who had less accurate knowledge; Participants who had a more positive attitude were 4 times more likely to have more frequent practice than those who had less positive attitude (AOR = 4.00, 95% CI = 2.44-6.56, P < 0.001). Moreover, the participants who had very less fear were 56% less likely to have more positive attitude (AOR = 0.44, 95% CI = 0.23-0.84, P < 0.01) and at the same time 53% less likely to have more frequent practice (AOR = 0.47, 95% CI = 0.26-0.84, P < 0.01) than those who had ~~high to moderate~~ fear (Table 5).

Table 4

Association of socio-demographic characteristics with knowledge, attitude, fear, and Practice score.

**Socio-demographic characteristics (n)**	**Knowledge**		**Attitude**		**Fear**		**Practice**	
	Mean Rank	U/H test P-value	Mean Rank	U/H test P-value	Mean Rank	U/H test P-value	Mean Rank	U/H test P-value
**Gender***								
Male (285)	195.32	0.225	186.58	0.130	176.37	**<0.001**	175.86	**<0.001**
Female (97)	180.26		205.95		235.96		237.46	
**Education***								
HSC (104)	183.24	0.349	200.50	0.323	204.98	0.144	206.07	0.112
BSc/MSc/Above(278)	194.59		188.13		186.46		186.05	
**Occupation***								
Student and self-employed (152)	197.62		177.89		181.38		180.61	

Student only (230)	187.45	0.357	200.49	**0.047**	198.19	0.144	198.70	0.115
**Family type***								
Nuclear (293)	188.11	0.225	187.76	0.224	188.37	0.313	194.41	0.347
Extended (89)	202.65		203.80		201.81		181.93	
**Income****								
<20,000 BDT (222) **(a)**	187.35	0.647	179.91	**0.05**	194.48	0.622	173.86	**0.001**
20,000-40,000 BDT (126) **(b)**	196.30		207.26		183.97		212.09	
>40,000 BDT (34) **(c)**	200.82		208.76		199.97		230.37	
**Location****								
Village (142) **(d)**	182.30	0.360	191.30	0.425	183.32	0.484	166.96	**0.004**
Sub-district town (39) **(e)**	210.58		197.91		182.69		189.85	
District town (97) **(f)**	187.76		203.31		204.63		217.15	
Divisional district town (104)	200.39		178.35		193.73		201.70	

BDT - Bangladesh Taka (Currency symbol of Bangladesh taka), HSC = Higher Secondary School Certificate, BSc- Bachelor of Science, MSc- Masters of Science, p- Significance value, *U = Mann-Whitney U, **H = Kruskal-Wallis H

[**(a)** vs **(b)** and **(a)** vs **(c)** were significant at 0.005 and 0.015 level respectively in practice (Bonferroni corrected)**;** **(a)** vs **(b)** was significant at 0.024 level in attitude (without Bonferroni correction)**;** **(d)** vs **(e)** was significant at 0.003 in practice (Bonferroni corrected)**;** **(d)** vs **(f)** was significant at 0.014 level in practice (without Bonferroni correction)]

Table 5

Results of multiple logistic regression on factors associated with knowledge, attitude, practice, and fear. (Adjusted model)

**Variables (D)**	**More Accurate knowledge**		**More Positive attitude**		**More frequent practice**		**Very little fear**	
	n (%)	AOR (95% CI)	n (%)	AOR (95% CI)	n (%)	AOR (95% CI)	n (%)	AOR (95% CI)
**Gender**								
Male (285)	243 (85.3)	1.14 (0.56-2.32)	182 (63.9)	1.22 (0.67-2.20)	140 (49.1)	**0.51* (0.29-0.89)**	232 (81.4)	**1.89* (1.05-3.40)**
Female (97)	81 (83.5)	ref	70 (72.2)	ref	69 (71.1)	ref	65 (67.0)	ref
**Education**								
HSC (104)	91 (87.5)	ref	74 (71.1)	ref	62 (59.6)	ref	79 (76.0)	ref
BSc/MSc/Above (278)	233 (83.8)	0.73 (0.36-1.49)	178 (64.0)	0.83 (0.47-1.45)	147 (52.9)	0.94 (0.55-1.62)	218 (78.4)	0.96 (0.53-1.74)
**Occupation**								
Student and self-employed (152)	130 (85.5)	ref	93 (61.2)	ref	74 (48.7)	ref	124 (81.6)	ref
Student only (230)	194 (84.3)	0.90 (0.47-1.73)	159 (69.1)	1.09 (0.65-1.84)	135 (58.7)	1.20 (0.73-1.99)	173 (75.2)	0.87 (0.48-1.58)
**Family type**								
Nuclear (293)	246 (84.0)	ref	189 (64.5)	ref	163 (55.6)	ref	230 (78.5)	ref
Extended (89)	78 (87.6)	1.45 (0.70-3.01)	63 (70.8)	1.28 (0.73-2.25)	46 (51.7)	0.86 (0.50-1.46)	67 (75.3)	0.73 (0.40-1.33)
**Income**								
<20,000 BDT (222)	184	0.89	135	**0.33***	103	0.63	177	0.53

	(82.9)	(0.27-3.0)	(60.8)	**(0.11-0.94)**	(46.4)	(0.26-1.55)	(79.7)	(0.19-1.40)
20,000-40,000 BDT (126)	110 (87.3)	1.09 (0.31-3.78)	89 (70.6)	0.41 (0.14-1.19)	81 (64.3)	1.06 (0.42-2.65)	93 (73.8)	0.50 (0.19-1.33)
>40,000 BDT (34)	30 (88.2)	ref	28 (82.4)	ref	25 (73.5)	ref	27 (79.4)	ref
**Location**								
Village (142)	113 (79.5)	ref	92 (64.8)	ref	63 (44.4)	ref	115 (81.0)	ref
Sub-district town (39)	37 (94.9)	**4.85* (1.07-22.05)**	26 (66.7)	0.844 (0.37-1.93)	19 (48.7)	0.98 (0.44-2.19)	31 (79.5)	0.92 (035-2.40)
District town (97)	83 (85.6)	1.46 (0.69-3.08)	71 (73.2)	0.99 (0.53-1.89)	63 (64.9)	**2.01* (1.09-3.65)**	72 (74.2)	0.78 (0.39-1.54)
Divisional district town (104)	91 (87.5)	2.03 (0.93-4.40)	63 (60.6)	**0.50* (0.27-0.93)**	64 (61.5)	**2.04* (1.12-3.73)**	79 (76.0)	0.66 (0.33-1.31)
**Knowledge**								
More accurate knowledge (324)	-	-	222 (68.5)	**2.34** (1.23-4.47)**	179 (55.2)	0.94 (0.49-1.80)	257 (79.3)	**2.17* (1.10-4.26)**
Less accurate knowledge (58)	-	-	30 (51.7)	ref	30 (51.7)	ref	40 (69.0)	ref
**Attitude**								
More positive attitude (252)	222 (88.1)	**2.39** (1.26-4.54)**	-	-	168 (66.7)	**4.00*** (2.44-6.56)**	183 (72.6)	**0.43** (0.22-0.82)**
Less positive attitude (130)	102 (78.5)	ref	-	-	41 (31.5)	ref	114 (87.7)	ref
**Practice**								
More frequent practice (209)	179 (85.6)	0.98 (0.51-1.88)	168 (80.4)	**4.01*** (2.44-6.55)**	-	-	146 (69.9)	**0.46** (0.26-0.83)**
Less frequent practice (173)	145 (83.8)	ref	84 (48.6)	ref	-	-	151 (87.3)	ref
**Fear**								
High to moderate fear (85)	67 (78.8)	ref	69 (81.1)	ref	63 (74.1)	ref	-	-
Very little fear (297)	257 (86.5)	**2.25* (1.13-4.47)**	183 (61.6)	**0.44** (0.23-0.84)**	146 (49.2)	**0.47** (0.26-0.84)**	-	-
**Total**	324 (84.8%)		252 (66.0%)		209 (54.7%)		297 (77.7%)	

D = Number of respondents in each category of independent variables, n = Number of respondents in category of dependent variables, BSc- Bachelor of Science, MSc- Masters of Science, AOR = Adjusted Odd ratio, Reference category = ref,
*P ≤ 0.05, **P ≤ 0.01, ***P ≤ 0.001, High to moderate fear = equal or more than 50% fear score, Very less fear = less than 50% fear score.

Table 6

Results of spearman's rank-order correlation among knowledge, attitude, practice, and fear score.

	1	2	3	4
**1**. Knowledge	1			
**2**. Attitude	0.12^{a} 0.015^{b}	1		
**3**. Practice	0.05^{a} 0.369^{b}	0.36^{a} $< 0.001^{b}$	1	
**4**. Fear	-0.05^{a} 0.280^{b}	0.24^{a} $< 0.001^{b}$	0.15^{a} 0.005^{b}	1

a = Correlation coefficient, b = Significance level (Two tailed)

Probable reasons explaining why students are detached from maintaining CPMs

When ~~asked to observe participants' thoughts as to~~ whether they are maintaining safety rules for Covid-19, 27% (103) participants assured that they are maintaining safety rules completely while 73% (279) reported "No" and mentioned their possible reasons ~~why~~ they were detached from maintaining safety rules properly. Among ~~those who responded "No",~~ 56.3% participants mentioned they cannot maintain a social distance because they need to go out for many reasons and need to use public transports where social distance maintaining is not possible while 44.8% participants mentioned they need to go market for daily groceries or ~~many~~ things where they cannot maintain social distance. 28.3% of the participants think of themselves as social butterflies and meet with many friends while they do not maintain social distance. 22.2% of the participants reported that they can't wash their hands regularly while 32.0% mentioned that ~~very few times a day,~~ they wash their hands as recommended by WHO. ~~The~~ further analysis explored that among participants who mentioned they wash their hands regularly, 22.1% do not wash their hands maintaining rules recommended by WHO ~~while 77.8% wash their hands regularly as recommended by WHO and~~ among those who mentioned they do not wash their hands regularly, 66.1% maintain rules recommended by WHO when they wash their hands. In addition, 37.6% of participants ~~don't~~ use hand sanitizers when staying outside the home and 23.3% of participants have bad habits of touching eyes, nose,

mouth, frequently. 21.1% of the participants reported that they have not seen anyone from their ~~relatives or relatives of relatives~~ or neighbors ~~got~~ infected by a coronavirus and that is why they do not give importance to maintaining safety rules of Covid-19. (Fig 2) depicted all the other reasons graphically with the frequency of responses for each reason and (Table 7) tabulated all the supportive information.

Fig 2 Graphical representation of reasons behind failure to maintain safety guidelines of covid-19. (Table 7) tabulated all the supportive information.

Table 7

Possible reasons why people cannot maintain safety rules of Covid-19 completely.

▪ Beliefs that I will not be infected by the coronavirus. **(C01)**
▪ Things related to corona seem to me as media-created rumors. **(C02)**
▪ No one having been identified as corona virus-positive yet from my relatives or relatives of relatives or neighbors makes maintaining safety rules less important to me. **(C03)**
▪ I am less panicked about coronavirus. **(C04)**
▪ I have no clear conception about safety rules for Covid-19. **(C05)**
▪ It is not always possible for me to wash hands coming from outsides. **(C06)**
▪ Very few times in a day, hands are washed up to the elbow for (20-30) min as recommended by WHO. **(C07)**
▪ I do not use hand sanitizer when staying outside. **(C08)**
▪ Trying but I can't give up the bad habit of touching eyes, face, nose frequently. **(C09)**
▪ I am to go to the market often for daily groceries or other things where maintaining social distance is not possible. **(C10)**
▪ I am to go out for many reasons and use public transports where maintaining social distance is not possible. **(C11)**
▪ I am a social butterfly and hang out with friends very often while social distance is not maintained. **(C12)**
▪ I don't maintain social distance thinking about the benefit of maintaining social distance when people around me don't maintain social distance. **(C13)**
▪ I don't use medical masks because of having any confidence in a normal medical mask (5 BDT) as well as no money for buying expensive masks**. (C14)**
▪ When wearing a mask, I feel very uneasy and it's very hard to stay with. **(C15)**
▪ I don't use a mask because it looks ugly. **(C16)**
▪ I don't know any reason. **(C17)**

BDT-Bangladesh Taka (Currency symbol of Bangladeshi taka)

Discussion

~~To fulfill the goals of this present study, several statistical analyses had been conducted and their outcomes had been presented sequentially in the result section. In this section, these outcomes have been discussed collaboratively with other previously published studies.~~

Knowledge level of Participants

The findings showed that participants' overall correct answer rate was 89.6%, indicating participants had appreciable ~~K~~nowledge on Covid-19. This outcome ~~was~~ consistent with ~~some~~ previous studies conducted last year (2020) in Bangladesh (80.8%) (18), China (90%) (19), Malaysia (80.5%) (20), Saudi Arabia (81.6%) (21), Iran (90% & 85%) (22), Nepal (60% to 98.7%) (23). Moreover, 84.8% of participants had more accurate knowledge which ~~was larger~~ compared to that reported by some previous Bangladeshi investigations (7–9,11,13), indicating knowledge level has elevated among Bangladeshis.

~~Our study reported that~~ knowledge scores do not significantly vary with gender, education, occupation, location, income, family type, which ~~was~~ inconsistent with some studies (7–9,11,13,19,24).

Attitude level of Participants

Approximately 66% of participants had a more positive attitude towards Covid-19, which was commensurate with an article (11). But when compared to the other two Bangladeshi articles, it seems attitude level among students has alleviated to some extent (8,9). Compared to overseas investigations, our finding was consistent with a study from Pakistan (65.4%) (12), while inconsistent with studies from Saudi Arabia (95%) (25), China (73.8%) (10). Our findings suggested that Bangladeshi students might have lost their confidence towards controlling the rapid escalation of coronavirus as only 35.3% of participants took it confidently that Bangladesh can win the battle against Covid-19 and concomitantly, only 44.2% of participants were confident that Covid-19 will finally be controlled. Last year, Rabbani et al., (2020) reported that 55.3% of their participants were confident about Bangladesh would win the battle against Covid-19 which was also consistent with Kundu et al., n.d. (2020), and 68.5% were confident that Covid-19 would finally be controlled which was somewhat lower compared to Banik et al., (2020) (9,13,26). On the other hand, a Malaysian, Chinese, Saudi Arabian, Indonesian, Nepali article published last year (2020) reported that they respectively obtained 83.1%, 90.8%, 94%, 94.3%, and 71.5% positive responses regarding whether Covid-19 would successfully be controlled, and 95.9%, 97.1%, 97%, 95.5%, and 80% positive response regarding whether their country would be able to win the battle against Covid-19 (19–

21,24,27). On the whole, it is obtrusive that attitude level among students were noticeably subsided. The reason behind obtaining such ominous outcomes might be the long duration of uncertainties which ~~has been~~ appeared due to ~~emerging~~ of new variants of SARS-COV-2 with the function of time.

Furthermore, our study explored that attitude levels significantly ~~vary~~ with occupation, and family income of the participants, not with other socio-demographic variables included in our scrutiny, which was somewhat ~~inconsistent~~ with several studies. (7,9,11).

Fear level of Participants

On average, participants had a 35.8% fear score which ~~was~~ lower compared to Hossain ~~MA,~~ et al.~~, (2020)~~ (28). Besides, only 2.1% of participants had very high fear ~~while~~ 77.7% had very less fear. These outcomes indicate that ~~Bangladeshi~~ students are no longer panicked. The fear score was significantly associated with ~~only gender~~ of the participants. Females had significantly high fear than males. Hossain ~~MA,~~ et al., ~~(2020)~~ reported that the fear score significantly differs with ~~gender~~, education, and geography, when female, urban-dwelling, and higher educated participants had high fear, which ~~was~~ partially similar to our findings (28).

Practice level of Participants

On average, participants had ~~a~~ 74.2% practice score and 55% participants had more frequent practice, which ~~was~~ consistent with some studies from Bangladesh (7,9,11,26). So, our ~~study~~ indicate~~s~~ that the preventive practice level remains similar to what was in last year. However, compared ~~to a study from~~ Bangladesh (24%) (13) and ~~from~~ Pakistan (36.5%) (12), our participants had more frequent practice ~~but seems to have much lower frequent practice when compared to the studies from Saudi Arab (81% & 81.9%) (14,25) and South Korea (87.9%) (10).~~ Indeed, our study found that ~~more frequent practice~~ is greatly influenced by ~~gender~~, family income and location. However, the ~~more frequent practice~~ could also be influenced by other socio-demographic factors such as education, occupation, age, etc. (8,9,11,13).

Relationship among knowledge, attitude, practice, and fear

The finding showed knowledge has an indirect effect on preventive practice mediated by attitude as knowledge has a positive impact on attitude and concomitantly, ~~an~~ attitude ~~has a positive impact on~~ preventive practice, ~~indicating the more knowledge on Covid-19, the more attitude towards it, and the more frequent practice towards its prevention.~~ Similar outcomes were reported in ~~some~~ other studies (8,13,15,25,29,30). Moreover, our study uncovered that knowledge has a significant inverse effect on fear, indicating the more accurate knowledge, the less fear. This outcome ~~was parallel with some other studies~~ (18,25,28,31). Our study

also revealed that like attitude, fear has also a positive impact on preventive practice, indicating the more fear, the more preventive practice. A similar outcome was reported by Hossain MA, et al,.(2020) & W/Mariam TG, et al,.(2020) (28,32). Ostensibly, this outcome could seem controversial but it represents the significant influence of fear on changing someone's behavior or attitude towards preventive practice. Our study also substantiates the immediate statement reporting that fear has a positive and mysteriously similar impact on attitude as was on preventive practice. However, we cannot expect high fear among the populace to have more practice because fear has some shortcomings too. For instance, Covid-19 fear is significantly associated with anxiety and psychological distress, which could in turn trigger stress and mental illness (31). Besides, there is an instance of suicidal death due to the fear of Covid-19 in Bangladesh (28,33). (Fig 3) depicted all the factors and their impact on preventive practice.

Fig 3 Diagram showing the direct and indirect impact of knowledge, attitude, fear, and socio-demographic factors (Ellipse) on preventive practice.

In addition, Our study reported that the majority of the participants thought they cannot adhere to preventive measures because of not maintaining social distance properly, followed by the reluctance in hand washing as recommended by WHO. We had no sufficient data to support these reasons.

Limitations, Strength, and Recommendation

Our study had several limitations. First, our study sample could not reflect all students throughout the country. Second, our study could not include students of all ages. Third, the study sample size was comparatively small. Fourth, our study followed a web-based cross-sectional study that excluded underprivileged students especially those who were not accessible to internet-based facilities. Five, we had to conduct comparatively less sensitive statistical analysis as our data were not normally distributed. As to the strength of our study, to the best of our knowledge, this is the first study that attempted to find out possible reasons why students are deflected from maintaining preventive measures. Future investigations should follow both community-based and web-based cross-sectional studies, collecting data from all sectors, all divisions, attempting to find out the most probable reasons behind failure to maintain all CPMs in a more structured way.

Conclusion

Our study attempted to bring up the present knowledge, attitude, practice, and fear level of Bangladeshi students. According to our study, students had appreciable knowledge and very little fear but disappointedly had average attitude and practice towards CPMs. Besides, students ~~had a lack of confidence on~~ Bangladesh ~~would~~ win the battle against Covid-19. Therefore, it is time for public health policymakers to

be more focused to scale up ~~students' confidence and attitude towards Covid-19~~. To do so, they must be more dogmatic to develop rules and regulations and more austere in implementing and maintaining those to combat any new onslaught of the deadly coronavirus. Besides, providing public health educations, arranging viable public health campaigns, promoting accurate information regarding Covid-19, counseling students as well as general people ~~with regards to~~ preventive practices, must be continued till the end of this unprecedented condition.

Supporting Information

S 1 File. Questionnaire and Coding information. (DOCX)

S 2 File. Covid-19 Raw Data. (XLSX)

Acknowledgments

The authors acknowledge all the participants who participated in the study and all the volunteers who helped in data collection. The authors are also grateful to prof. Dr. Md. Golam Sadik & Dr. Md. Abdul Rafi for their support in the overall improvement of the manuscript.

Author contributions

Conceptualization: Tahsin Ahmed Rupok, Sunandan Dey

Data curation: Tahsin Ahmed Rupok, Rashni Agarwala, Md. Nurnobi Islam, Sunandan Dey, Bayezid Bostami.

Formal Analysis: Tahsin Ahmed Rupok, Rashni Agarwala.

Investigation: Tahsin Ahmed Rupok, Rashni Agarwala, Md. Nurnobi Islam, Sunandan Dey.

Methodology: Tahsin Ahmed Rupok, Bayezid Bostami.

Project Administration: Tahsin Ahmed Rupok, Sunandan Dey.

Supervision: Tahsin Ahmed Rupok, Sunandan Dey.

Writing – original draft: Tahsin Ahmed Rupok.

Writing – review & editing: Tahsin Ahmed Rupok, Md. Nurnobi Islam, Sunandan Dey, Rashni Agarwala, Bayezid Bostami.

Reference

1. Correspondent S. Country logs highest Covid cases in a day. Daily Star [Internet]. 2021 Jul 1; Available from: https://www.thedailystar.net/news/bangladesh/news/country-logs-highest-covid-cases-day-2121453

2. Correspondent S. 112 more die, 7,666 get Covid. Daily Star [Internet]. 2021

Jun 30; Available from: https://www.thedailystar.net/bangladesh/news/112-more-die-7666-get-covid-2120757

3. SARS-CoV-2 Delta variant [Internet]. Available from: https://en.wikipedia.org/wiki/SARS-CoV-2_Delta_variant

4. Bangladesh could face severe Covid-19 surge by June-end if Indian variant spreads: experts. Daily Star [Internet]. 2021 May 25; Available from: https://www.thedailystar.net/coronavirus-deadly-new-threat/news/bangladesh-could-face-severe-covid-19-surge-june-end-if-indian-variant-spreads-experts-2098725

5. Majumdar BA. How do we tackle this new onslaught of the coronavirus? Daily Star [Internet]. 2021 Jun 28; Available from: https://www.thedailystar.net/opinion/news/how-do-we-tackle-new-onslaught-the-coronavirus-2119313

6. Correspondent S. Lockdown now from Thursday. Daily Star [Internet]. 2021 Jun 27; Available from: https://www.thedailystar.net/frontpage/news/lockdown-now-thursday-2119017

7. Paul A, Sikdar D, Hossain MM, Amin MR, Deeba F, Mahanta J, et al. Knowledge, attitudes, and practices toward the novel coronavirus among Bangladeshis: Implications for mitigation measures. PLoS One [Internet]. 2020;15(9 September 2020):1–18. Available from: http://dx.doi.org/10.1371/journal.pone.0238492

8. Hossain MB, Alam MZ, Islam MS, Sultan S, Faysal MM, Rima S, et al. Do knowledge and attitudes matter for preventive behavioral practices toward the COVID-19? A cross-sectional online survey among the adult population in Bangladesh. Heliyon. 2020;6(12). Available from: https://doi.org/10.1016/j.heliyon.2020.e05799

9. Banik R, Rahman M, Sikder M, Rahman QM, Pranta MUR. Investigating knowledge, attitudes, and practices related to COVID-19 outbreak among Bangladeshi young adults: A web-based cross-sectional analysis. J public Heal [Internet]. 2020 [cited 2021 Mar 31]; Available from: https://doi.org/10.1007/s10389-020-01432-7

10. Peng Y, Pei C, Zheng Y, Wang J, Zhang K, Zheng Z, et al. A cross-sectional survey of knowledge , attitude and practice associated with COVID-19 among undergraduate students in China. BMC Public Health [Internet]. 2020; Available from: https://doi.org/10.1186/s12889-020-09392-z

11. Ferdous MZ, Islam MS, Sikder MT, Mosaddek ASM, Zegarra-Valdivia JA, Gozal D. Knowledge, attitude, and practice regarding COVID-19 outbreak in Bangladesh: An onlinebased cross-sectional study. PLoS One [Internet]. 2020;15(10 October):1–17. Available from: http://dx.doi.org/10.1371/journal.pone.0239254

12. Salman M, Ul Z, Noman M, Abbas H, Khalid Z. Knowledge , attitude and preventive practices related to COVID ‐ 19 : a cross ‐ sectional study in two Pakistani university populations. Drugs Ther Perspect [Internet]. 2020;36(7):319–25. Available from: https://doi.org/10.1007/s40267-020-00737-7

13. Rabbani MG, Akter O, Hasan MZ, Samad N, Mahmood SS, Joarder T. Knowledge, Attitude and Practice towards COVID-19 among people in Bangladesh during the pandemic: a cross-sectional study. medRxiv [preprint] [Internet]. 2020; Available from: https://doi.org/10.1101/2020.09.22.20198275

14. Alhazmi A, Hamed M, Ali M, Mohieldin A, Aziz F, Osman OB, et al. Knowledge , attitudes and practices among people in Saudi Arabia regarding COVID-19 : A cross-sectional study. J Public health Res [Internet]. 2020;9(3):345–53. Available from: https://www.ncbi.nlm.nih.gov/pmc/articles/PMC7520855/

15. Lee M, Kang BA, You M. Knowledge, attitudes, and practices (KAP) toward COVID-19: a cross-sectional study in South Korea. BMC Public Health. 2021;21(1):1–10. Available from: https://doi.org/10.1186/s12889-021-10285-y

16. Tayeb T. Bangladesh's Covid lockdown conundrum. Daily Star [Internet]. 2021 Apr 18; Available from: https://www.thedailystar.net/opinion/closer-look/news/bangladeshs-covid-lockdown-conundrum-2079065?amp

17. Ahorsu DK, Lin C, Imani V, Saffari M, Griffiths MD, Pakpour AH. The Fear of COVID-19 Scale : Development and Initial Validation. Int J Ment Health Addict [Internet]. 2020; Available from: https://doi.org/10.1007/s11469-020-00270-8

18. Ali M, Uddin Z, Banik PC, Hegazy F, Zaman S, Ambia ASM, et al. Knowledge, attitude, practice and fear of COVID-19: A cross-cultural study. medRxiv [preprint]. 2020;1–27. Available from: https://doi.org/10.1101/2020.05.26.20113233

19. Zhong B, Luo W, Li H, Zhang Q, Liu X, Li W, et al. Knowledge , attitudes ,

and practices towards COVID-19 among Chinese residents during the rapid rise period of the COVID-19 outbreak : a quick online cross-sectional survey. Int J Biol Sci. 2020;16(10). Available from: https://doi.org/10.7150/ijbs.45221

20. Azlan AA, Hamzah MR, Jen T, Id S, Hadi S, Id A. Public knowledge , attitudes and practices towards COVID-19 : A cross-sectional study in. PLoS One [Internet]. 2020;15(5):1–15. Available from: http://dx.doi.org/10.1371/journal.pone.0233668

21. Al-Hanawi MK, Angawi K, Alshareef N, Qattan AMN, Helmy HZ, Abudawood Y, et al. Knowledge, Attitude and Practice Toward COVID-19 Among the Public in the Kingdom of Saudi Arabia: A Cross-Sectional Study. Front Public Heal. 2020;8(May):1–10. Available from: https://doi.org/10.3389/fpubh.2020.00217

22. Erfani A, Shahriarirad R, Ranjbar K, Mirahmadizadeh A, Moghadami M. Knowledge, attitude and practice toward the novel coronavirus (COVID-19) outbreak- A population-based survey in Iran. Bull World Health Organ [Internet]. 2020; Available from: http://dx.doi.org/10.2471/BLT.20.256651

23. Hussain A, Garima T, Singh BM, Ram R, Tripti RP. Knowledge, attitudes, and practices towards COVID-19 among Nepalese Residents: A quick online cross-sectional survey. Asian J Med Sci. 2020;11(3):6–11. Available from: https://doi.org/10.3126/ajms.v11i3.28485

24. Paudel S, Shrestha P, Karmacharya I, Pathak OK. Knowledge, attitude, and practices (KAP) towards COVID-19 among Nepalese residents during the COVID-19 outbreak: An online cross-sectional study. 2020; Available from: https://doi.org/10.21203/rs.3.rs-31044/v1

25. Alahdal H, Basingab F, Alotaibi R. An analytical study on the awareness, attitude and practice during the COVID-19 pandemic in Riyadh, Saudi Arabia. J Infect Public Health [Internet]. 2020;13(10):1446–52. Available from: https://doi.org/10.1016/j.jiph.2020.06.015

26. Kundu S, Sayeed A, Banna MH Al, Begum MR, Kormoker T, Brazendale K, et al. Knowledge, attitudes, and practices towards measures for prevention of the spread of COVID-19: An online cross-sectional survey among Bangladeshi residents. PsyArxiv [Preprints] [Internet]. :1–22. Available from: https://doi.org/10.31234/osf.io/cxp8n

27. Muslih M, Susanti HD, Rias YA. Knowledge , Attitude , and Practice of Indonesian Residents toward COVID-19 : A Cross-Sectional Survey. Int J

Environ Res Public Health [Internet]. 2021; Available from: https://doi.org/10.3390/ijerph18094473

28. Hossain MA, Hossain KMA, Walton LM, Uddin Z, Haque MO, Kabir MF, et al. Knowledge, attitudes, and fear of COVID-19 during the rapid rise period in Bangladesh. medRxiv [Internet]. 2020 [cited 2021 Mar 31]; Available from: https://journals.plos.org/plosone/article?id=10.1371/journal.pone.0239646

29. Lau LL, Hung N, Go DJ, Ferma J, Choi M, Dodd W, et al. Knowledge, attitudes and practices of COVID-19 among income-poor households in the Philippines: A cross-sectional study. J Glob Health. 2020;10(1). Available from: https://doi.org/10.7189/JOGH.10.011007

30. Gao H, Hu R, Yin L, Yuan X, Tang H, Luo L, et al. Knowledge, attitudes and practices of the Chinese public with respect to coronavirus disease (COVID-19): An online cross-sectional survey. BMC Public Health. 2020;1–8. Available from: https://doi.org/10.21203/rs.3.rs-40292/v1

31. Saravanan C, Mahmoud I, Elshami W, Taha MH. Knowledge, Anxiety, Fear, and Psychological Distress About COVID-19 Among University Students in the United Arab Emirates. Front Psychiatry. 2020;11(October):1–10. Available from: https://doi.org/10.3389/fpsyt.2020.582189

32. W/Mariam TG, Kassie BA, Asratie MH, Abate AT. The Effects of Fear and Knowledge of COVID-19 on Preventive Practice Among Pregnant Women Who Attend Antenatal Care in Northwest Ethiopia, 2020: Institution-Based Cross-Sectional Study. Int J Womens Health. 2021;Volume 13:95–100. Available from: https://doi.org/10.2147/ijwh.s286088

33. Mamun MA, Gri MD. First COVID-19 suicide case in Bangladesh due to fear of COVID-19 and xenophobia : Possible suicide prevention strategies. Asian J Psychiatr. 2020;51(April):2–3. Available from: https://doi.org/10.1016/j.ajp.2020.102073

Fig 1. tif

Click here to access/download;Figure;Fig 1. tif.tif

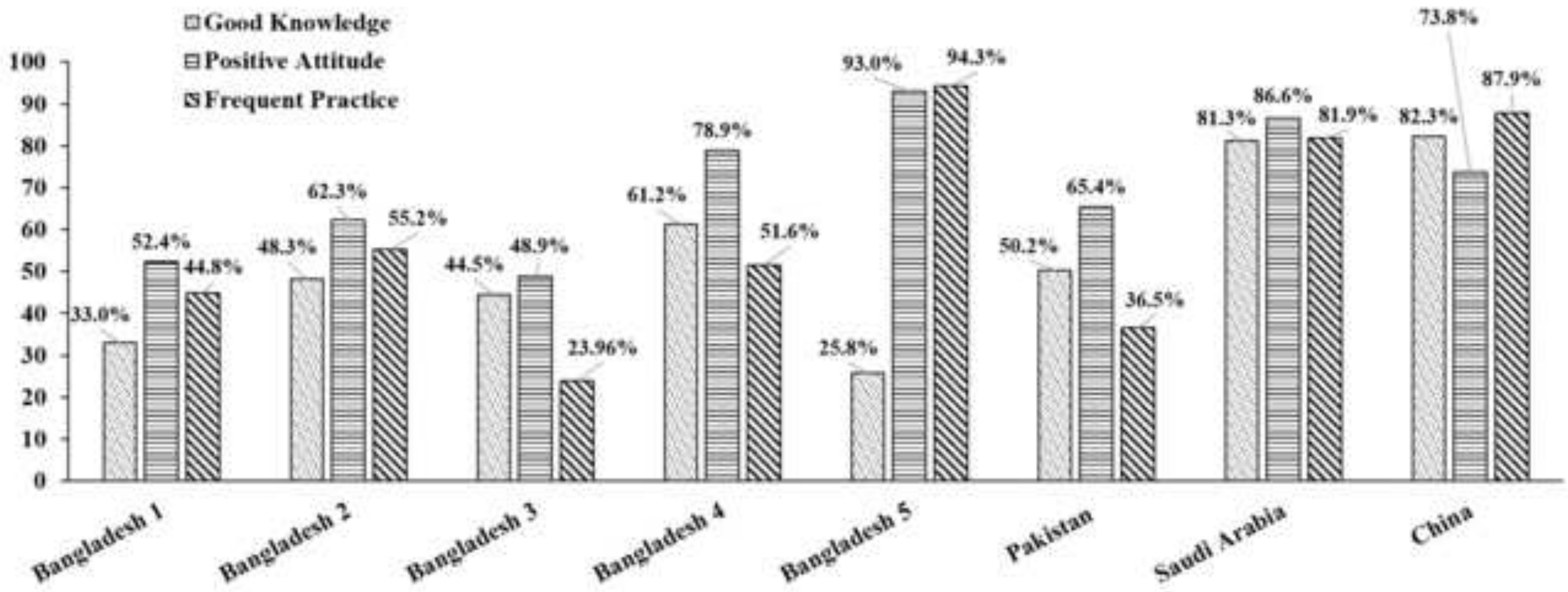

Click here to access/download;Figure;Fig 2. tif.tif

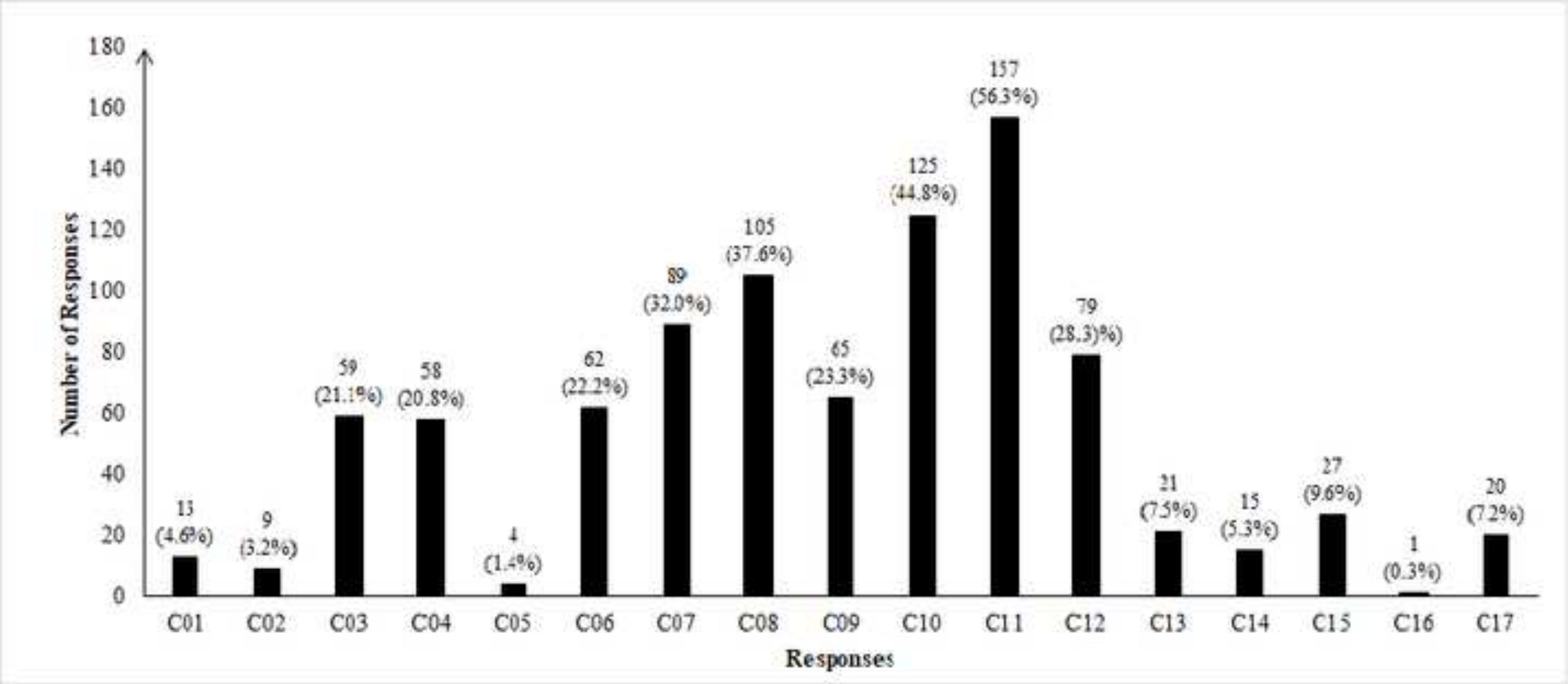

Fig 3. tif

Click here to access/download;Figure;Fig 3. tif.tif

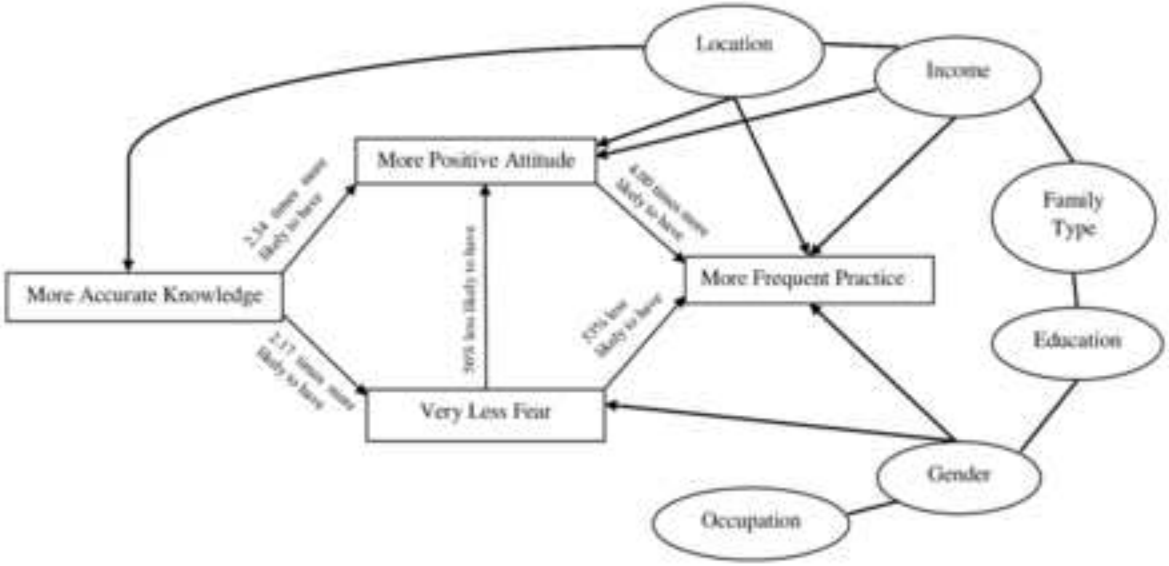

Click here to access/download
Supporting Information
S 1 File. Questionnaire and Coding information.docx

Click here to access/download
Supporting Information
S 2 File Covid-19 Raw Data.xlsx

Click here to access/download
Supporting Information
Approval letter.jpg

Knowledge, attitude, practice, and fear level of Bangladeshi students towards Covid-19 after a year of the pandemic situation: a web-based cross-sectional study.

Short title: Knowledge, attitude, practice, and fear level of Bangladeshi Students towards Covid-19.

Authors and affiliations:

1. Tahsin Ahmed Rupok**, Email: tahsinahmedrupokpharmru9176@gmail.com

Department of Pharmacy, University of Rajshahi, Rajshahi-6205, Bangladesh.

2. Sunandan Dey, Email: sunandandeypharmacy@gmail.com

Department of Pharmacy, University of Rajshahi, Rajshahi-6205, Bangladesh.

3. Rashni Agarwala, Email: rashniagarwala@gmail.com

Department of Pharmacy, Islamic University, Kushtia-7003, Bangladesh.

4. Md. Nurnobi Islam, Email: nurnobi6329@gmail.com

Department of Chemistry, Shahjalal University of Science & Technology, Sylhet-3114, Bangladesh.

5. Bayezid Bostami, Email: bayezidpharm.ru@gmail.com

Department of Pharmacy, University of Rajshahi, Rajshahi-6205, Bangladesh

**Corresponding author: tahsinahmedrupokpharmru9176@gmail.com

Abstract

In the earlier phase of the pandemic situation, the Government of Bangladesh (GoB) badly suffered to adhere their people to preventive measures probably due to less knowledge and attitude towards Covid-19. To tackle the second wave of coronavirus, GoB again enforced an array of preventive measures, but still encountering the same problem after a year of the pandemic situation. As an attempt to find out the reasons behind this, our study aimed to assess the present knowledge, attitude, practice, and fear level of the students. A cross-sectional study was designed and conducted from 15th to 25th April 2021. A total of 382 participants met all the inclusion criteria and were considered for performing all the statistical analyses (Descriptive statistics, Mann-Whitney U test, Kruskal-Wallis H test, Multiple logistic regression, Spearman rank-order correlation). All the participants were students aged 16 to 30. 84.8%, 66%, 55%, and 22.3% participants had more accurate knowledge, more positive attitude, more frequent practice, and moderate to high fear towards Covid-19, respectively. Knowledge, attitude, practice, and fear were interrelated directly or indirectly. It was found knowledgeable participants were more likely to have more positive attitude (AOR = 2.34, 95% CI = 1.23-4.47, $P < 0.01$) and very little fear (AOR = 2.17, 95% CI = 1.10-4.26, $P < 0.05$). More positive attitude was found as a good predictor of more frequent practice (AOR = 4.00, 95% CI = 2.44-6.56, $P < 0.001$), and very less fear had negative impact on both attitude

(AOR = 0.44, 95% CI = 0.23-0.84, $P < 0.01$) and practice (AOR = 0.47, 95% CI = 0.26-0.84, $P < 0.01$). The findings reflect that students had appreciable knowledge and very little fear, but disappointedly had average attitude and practice towards Covid-19 preventive measures (CPMs). In addition, students had lack of confidence on Bangladesh would win the battle against Covid-19. Thus, our findings make a recommendation that policymakers should be more focused to scale up students' confidence and attitude towards CPMs by developing and implementing well-conceived plan of actions besides insisting them to practice CPMs.

Keywords: Knowledge; Attitude; Practice; Fear; Covid-19; Bangladesh.

Introduction

In Bangladesh, both infection and death rates have started to burgeon again from the second week of March 2021 with a small dip in late April, which has been further instigated due to the entrance of highly transmissible Delta variant of coronavirus in May 2021(1). As of June 29, 2021, 7,666 new cases were detected with a positivity rate of 23.97%, and 112 people lost their life (2). Delta variant, first detected in India in late 2020, is highly contagious and deadly (3). If this Delta variant starts to escalate vehemently in Bangladesh as it did in India and Nepal, people stay lackadaisical in maintaining public health hygiene, and governments continue to

develop an ill-conceived plan of action and fail to ensure proper implementation of preventive measures, then, the number of cases detected per day could rise to an uncontrollable digit, which can throw the situation into turmoil (4). So, the government should immediately take the necessary steps to prevent any grim situation. It is worth mentioning that prevention is the best and comparatively less costly method to the most effective protection against the virus. Only adequate preventive measures can keep the infection rate under control, which will truncate both the number of patients and death tallies. As a preventive measure, the government decided to go for countrywide strict lockdown again from 1st July 2021 but it is worth remembering that lockdowns are only effective if they are accompanied by other preventive measures (5,6). However, the GoB badly suffered to adhere their population to preventive measures in the earlier phase of the pandemic situation probably due to people's less knowledge and attitude to embrace the measures taken by the governments. A review of some previous studies conducted in Bangladesh reflected the truth that in last year (2020) Bangladeshi people were comparatively less knowledgeable, had a less positive attitude toward Covid-19, and as a consequence had less frequent practice, which was consistent with a study of Pakistan but inconsistent with China and Saudi Arabia (7–14). Indeed, there are also exceptions such as one article from Bangladesh (9) showed people (78.9%) had a comparatively more positive attitude towards Covid-19.

Moreover, another article from Bangladesh (8) reported that people had more frequent practice (94.3%) along with more positive attitude (93.0%) towards CPMs (Fig 1).

However, one thing to remember, adhering people to preventive measures is not straightforward, takes intensive efforts over a length of time as well as depends on what extent people can perceive the importance of those measures. So, proper implementation of these preventive measures warrants adequate well-planned initiatives to scale up people's knowledge and awareness towards Covid-19 and its preventive measures, which will ultimately alter their behavior as well as attitude positively towards Covid-19 preventive practice. Several studies published last year (2020) outlined that adequate knowledge regarding Covid-19 is a potential predictor of attitude towards its preventive practice (13,15). To speak the truth, it is indefinite when the coronavirus will go away from the world. Therefore, people must be resilient to this new situation, learn all fundamental information regarding Covid-19, and change their attitude positively towards practicing all safety guidelines stipulated by their government.

Fig 1 Diagram reflecting different countries and their peoples' knowledge, attitude, and practice level towards covid-19. ***[Bangladesh 1*** (7)***, Bangladesh 2*** (11)***, Bangladesh 3*** (13)***, Bangladesh 4*** (9)***, Bangladesh 5 (8), Pakistan (12), Saudi Arabia*** (14)***, China*** (10)***].***

However, since the emergence of the pandemic situation, the GoB have been confronting troubles to aware and adhere their people to CPMs. After a year of pandemic situation, the situation seems to remain stagnant and the GoB is still facing the same problem. Therefore, it is needed to explore the present KAP level of Bangladeshi people. Anyway, this study aimed to bring up the present KAP level of Bangladeshi students and how well-prepared they are to confront any future onslaught of Covid-19. So, the objectives of this present study were-

-To explore the present knowledge, attitude, practice, and fear level of the Bangladeshi students and their associated potential factors.

-To find out whether knowledge, attitude, practice, and fear are related to each other.

-To find out the most probable reasons why students are digressed from maintaining CPMs enforced by the governments.

Materials and Methods

Participants and Data recruitment procedure

This web-based cross-sectional survey was conducted on Bangladeshi students using online facilities amid the second wave of coronavirus in Bangladesh, continued for 10 days from 15th to 25th April in 2021. A structured questionnaire was prepared in a Google Form and a link was generated, shared with all authors and other volunteers who were instructed properly as to how well they can use this form to recruit data

with adequate consent from the participants and from the guardians of the participants aged under 18 years old. A face-to-face community-based survey was not possible as the GoB implemented their second strict lockdown from 14th April being one day ahead of our data collection initiated (16), all data were therefore assembled through online approaches using Whatsapp, Messenger, Email, Facebook. This survey included subjects aged 16 years old or above, being Bangladeshi residents, having internet access and ability to communicate, having no intellectual disability. Subjects living in foreign countries, having no or less interest to participate in were excluded from the survey. A total of 382 participants successfully submitted their data and all completed the questionnaire adequately as they were recommended.

Survey Instrument

The questionnaire was prepared after a thorough review of some literature published last year (2020), which had following six sections.

Preface

This section had a brief delineation of the objectives of the survey and some instructions on how to complete the questionnaire properly. It also included a question asked to take consent of the respondents.

Socio-demographic Measures

This section embodied 7 questions asked to gather socio-demographic information of a participant. Socio-demographic information included gender, age, education, occupation, location, family type, family income which was categorized as follows (<20,000 BDT), (20,000-40,000 BDT), (>40,000 BDT).

Knowledge Scale

This section had 12 questions where each question had three options "Right", "Wrong", and "Not sure". During scoring, responses of the questions with "Wrong" as a correct answer (Table 2) were first recoded from "Wrong" to "Right". Then, the correct answer (Right) was coded as 1 and the incorrect answer (Wrong/Not sure) was coded as 0. The total score ranged from 0 to 12 where those who obtained 83% score or greater (≥ 10) were considered to have more accurate knowledge otherwise considered having less accurate knowledge. Instead of considering 80% score for more accurate knowledge based on Bloom's cut off point, 83% score was considered to avoid fractional digits as all the knowledge scores were integer numbers.

Attitude Scale

This section had 8 questions where each question had three options "Yes", "No", and "Maybe" except one question having "Not possible" instead of "Maybe" in three options (Table 2). During scoring, four kinds of options were coded as follows, 0

(No/ Not possible for work), 1 (Maybe), and 2 (Yes). The total score ranged from 0 to 16. Those who had an 81% score or greater (≥ 13) were considered to have a positive attitude otherwise considered to have a less positive attitude. Instead of considering 80% score for positive attitude based on Bloom's cut off point, 81% score was considered to avoid fractional digits as all the attitude scores were integer numbers.

Practice Scale

This section also had 8 questions where each question had three options "Yes", "No", and "Sometimes". During scoring, three options were coded as follows, 0 (No), 1 (Sometimes), and 2 (Yes). The total score ranged from 0 to 16. Those who had an 81% score or greater (≥ 13) were considered to have more frequent practice otherwise considered having less frequent practice. Instead of considering 80% score for more frequent practice based on Bloom's cut off point, 81% score was considered to avoid fractional digits as all the practice scores were integer numbers.

Fear Scale

This section included a fear measuring tool which was reported as a valid and reliable instrument to assess Covid-19 related fear among respondents (17). This tool had 7 items where each item had five Likert options which were coded as follows, 0 (Strongly disagree), 1 (Disagree), 2 (Neutral), 3 (Agree), 4 (Strongly Agree). The

total score ranged from 0 to 28. Fear scores greater or equal to 21 were cut-points for high fear. Scores between 20-14 were cut points for moderate fear and scores equal to or less than 13 were cut points for less fear.

This section also had two extra questions. The first question having binary options “Yes”, “No” asked if or not they are maintaining preventative measures properly stipulated by the governments. The second question was asked to those who answered “No” to account for their reasons why they are aberrant than those who answered “Yes”. There were 17 possible responses and multiple responses could be selected.

Reliability & Validity

Reliability tests of the instrument were performed using field data to determine the validity and reliability of those scales mentioned above. Cronbach’s alpha for knowledge, attitude, practice, and fear scale were 0.47, 0.61, 0.81, and 0.83. Due to a shortage of time, we could not conduct a pilot study.

Ethical considerations

This study was conducted according to the principles expressed in the declaration of Helsinki. Besides, this study received ethical approval from Institute of Biological Science, University of Rajshahi, Bangladesh (ERC Memo no: 91/320IAMEBBC/IBSc). Anonymity and confidentiality were strictly maintained.

All respondents gave full informed consent to participate and consent for their data to be used in the publication. Participants aged under 18 years old were requested to provide their guardians’ Email id or Messenger id or Whatsapp id or Phone number so that authors or volunteers can contact their guardians to inform them about the objectives of the survey, and take consents from them regarding their children’s participation. This study included only those participants aged under 18 years old who made their guardians contact with authors or volunteers.

Data Management and Analysis

All submitted data were automatically stored in a dynamic Microsoft excel sheet which was made available offline after completion of data collection for primary data processing including data duplication-checking, data cleaning, data coding, etc. In this stage, three data fallen out of inclusion criteria of age (below 16 years old), and two data got duplicated were discard from the preliminary dataset. After accomplishment of data cleaning and coding, the final dataset having data of 382 respondents was entered into Statistical Package for the Social Science (SPSS) version 26.0 for further data processing and doing all the statistical analysis which included descriptive statistics (to determine percentage, mean, standard deviation), Mann-Whitney U test (to determine difference between two groups under the same

variable having two groups), Kruskal Wallis H test (to determine difference among the groups under same variable having more than two groups), Spearman rank-order correlation (if variables are ordinal), Multiple logistic regression (to determine predictors for accurate knowledge, positive attitude, frequent practice, very little fear) including gender, education, occupation, family type, family income, location, knowledge, attitude, practice, fear as independent variables. Non-parametric Mann-Whitney U test and Kruskal Wallis H test, and Spearman rank-order correlation were performed as the data under continuous dependent variables were not normally distributed. To check that, besides visual inspection of histograms, Shapiro-Wilk and Kolmogorov-Smirnov tests were conducted. Both tests were statistically significant at 0.01 for those continuous dependent variables and histograms of them looked left-skewed, which confirmed well that data under these variables were not normally distributed. For all statistical analyses noted above, the statistical significance level was set to 0.05 (Alpha value).

Results

Demographic characteristics of the sample

This study considered a total of 382 participants for final statistical analysis. Among them majority were male (74.6%), studying in BSc and MSc (71.8%). All the participants were students while 39.8% were self-employed. About 76.7% and

58.1% of the participants were from nuclear and lower income family (<20,000 BDT) respectively. 37.2% of the participants informed their location village. This study inclined towards the adult population as all participants had ages of 16 to 30 years old. (Table 1) tabulated detailed information about the socio-demographic characteristics of the respondents.

Table 1

Socio-demographic characteristics of the respondents.

Characteristics		Frequency	Percentage (%)
Gender	Male	285	74.6
	Female	97	25.4
Education	HSC	104	27.2
	BSc/MSc/Above	278	71.8
Occupation	Student and self-employed	152	39.8
	Student only	230	60.2
Family type	Nuclear	293	76.7
	Extended	89	23.3
Income	<20,000 BDT	222	58.1
	20,000-40,000 BDT	126	33.0
	>40,000 BDT	34	8.9
Location	Village	142	37.2
	Sub-district town	39	10.2
	District town	97	25.4
	Divisional district town	104	27.2

HSC- Higher Secondary School Certificate, BSc- Bachelor of Science, MSc- Masters of Science

Knowledge and its factors

Participants' Overall correct answer rate was 89.6% [Mean Score (MS) ± Standard Deviation (SD) = 10.76±1.20 out of 12] indicating participants had appreciable knowledge on Covid-19. 84.8% of the participants had more accurate knowledge

while only 15.2% had less accurate knowledge. The distribution of responses for each question of the knowledge scale was presented in (Table 2). Mann-Whitney and Kruskal-Wallis tests showed that knowledge scores do not vary with any socio-demographic variables included in our study, indicating all the groups of a variable had quite similar knowledge on Covid-19 disease (Table 4). Multiple logistic regression analysis identified location, attitude, and fear as significant factors that can predict if an individual would have more accurate knowledge on Covid-19 rather than having less accurate knowledge (Table 5).

Attitude and its factors

On average, participants had an 82% score on the attitude scale (MS ± SD = 13.14 ± 2.21 out of 16).Approximately 66% of the participants had a more positive attitude. Only 35.3% of the participants had confidence that Bangladesh can win the battle against Covid-19 and only 44.2% agreed that Covid-19 will finally be controlled. The distribution of responses for each question of the attitude scale was presented in (Table 2). Mann-Whitney and Kruskal-Wallis tests showed attitude scores vary with occupation (P= 0.047) and social status (P= 0.05). It was found that the participants who are student and self-employed, and came from a lower income family (<20,000 BDT) had significantly less positive attitude than their counterparts. (Table 4). Multiple logistic regression analysis identified income, location, knowledge, practice, fear, as significant factors that can predict if an individual would have a

more positive attitude rather than a less positive attitude. It was found that the participants who came of a lower income family (<20,000 BDT) were 67% less likely to have more positive attitude than those who came of a higher income family (> 40,000 BDT) (AOR = 0.33, 95% CI = 0.11-0.94, P < 0.05) and the participants came from divisional district town were 50% less likely to have more positive attitude than those came from village (AOR = 0.50, 95% CI = 0.27-0.93, P < 0.05) (Table 5).

Practice and its factors

On average, participants had a 74.2% score on the practice scale (MS ± SD = 11.87 ± 3.80 out of 16). Approximately 55% of the participants had more frequent practice. The distribution of responses for each question of the practice scale was presented in (Table 2). Mann-Whitney and Kruskal-Wallis tests uncovered that Practice scores vary with gender (P < 0.001), income (P < 0.001), and location (P < 0.01). It was found that females had significantly higher practice score than males (P < 0.001); Participants came of both higher income family (P < 0.01) and middle income family (P < 0.01) had significantly higher practice score than those came of a lower income family; Participants currently living in any district town and sub-district town (P ≤ 0.01) had significantly higher score on practice scale than those living in a village (Table 4). Multiple logistic regression analysis identified gender, location, attitude, fear, as significant factors that can predict if an individual would have more frequent

practice rather than less frequent practice. It was found that males were less likely to have more frequent practice than females by 49% (AOR = 0.51, 95% CI = 0.29-0.89, $P \leq 0.05$). The participants living in any district town or any divisional district town were 2 times more likely to have more frequent practice than those living in a village (AOR = 2.01, 95% CI = 1.09-3.65, $P \leq 0.05$);(AOR = 2.04, 95% CI = 1.23-4.47, $P \leq 0.01$) (Table 5).

Table 2

Participants' knowledge, attitude, and practice towards covid-19 (N = 382) (Distribution of Responses)

Knowledge Questions (Correct Answer)	Right n (%)	Wrong n (%)	Not Sure n (%)
Covid-19 disease is deadly but preventable as well as curable. (Right)	363 (95)	7 (1.8)	12 (3.1)
Fever, fatigue, dry cough, and breathing difficulty are the main clinical symptoms of Covid-19. (Right)	357 (93.5)	11 (2.9)	14 (3.7)
Elderly people having chronic illnesses and other complications are more likely to be seriously affected. (Right)	370 (96.5)	2 (0.5)	10 (2.5)
This disease can be prevented with antibiotics and other medications. (Wrong)	89 (23.3)	189 (49.5)	104 (27.2)
A person with Covid-19 having no symptoms (fever) cannot spread the virus to others. (Wrong)	30 (7.9)	314 (82.2)	38 (9.9)
Coronavirus can spread via respiratory droplets (from coughing, sneezing) of infected people. (Right)	370 (96.9)	8 (2.1)	4 (1.0)
Taking measures is not necessary for children and young adults to prevent infections by the coronavirus. (Wrong)	7 (1.8)	360 (94.2)	15 (3.9)
Isolation and treatment of people with Covid-19 are effective ways to reduce the spread of the virus. (Right)	354 (92.7)	14 (3.7)	14 (3.7)
People who have contact with someone infected with the coronavirus or came from an infected area/country should be immediately isolated for 14 days. (Right)	374 (97.9)	4 (1.0)	4 (1.0)
Covering the mouth and nose with a bent elbow or tissue paper or handkerchief when coughing or sneezing can reduce the spread of this virus. (Right)	357 (93.5)	16 (4.2)	9 (2.4)
This disease could be asymptomatic. (Right)	328 (85.9)	23 (6.0)	31 (8.1)
Individuals should avoid going to crowded places such as markets and public transportations to prevent infection. (Right)	374 (97.9)	6 (1.6)	2 (0.5)
**Table 02 (Continued)**			
Attitude Questions	Yes n (%)	No n (%)	Maybe/Not Possible for work n (%)
Do you like to stay at home for a certain period (2 weeks) to prevent coronavirus spread if the government will order so? **	330 (86.4)	24 (6.3)	28 (7.3)

Do you think that social distancing (e.g. stay 1-2 m apart, avoid crowds, etc.) can prevent the spread of this virus?	320 (83.8)	14 (3.7)	48 (12.6)
Do you agree that we should cancel business/ recreational trips at this time?	249 (65.2)	84 (22.0)	49 (12.8)
Do you believe that working from home can help to control Covid-19?	284 (74.3)	40 (10.5)	58 (15.2)
Do you agree that government should have taken preventive measures when new variants were first reported in Bangladesh?	364 (95.3)	3 (0.8)	15 (3.9)
Do you think health education can play an important role in Covid-19 prevention?	357 (93.5)	7 (1.8)	18 (4.7)
Do you agree that Covid-19 will finally be successfully controlled?	169 (44.2)	28 (7.3)	185 (48.4)
Do you have confidence that Bangladesh can win the battle against the Covid-19 disease?	135 (35.3)	52 (13.6)	195 (51.0)
**Table 02 (Continued)**			
Practice Questions	Yes n (%)	No n (%)	Sometimes n (%)
Do you wash hands with water and soap frequently?	241 (63.1)	30 (7.9)	111 (29.1)
Do you always use a mask?	305 (79.8)	20 (5.2)	57 (14.9)
Do you maintain the rules of using a mask?	304 (79.6)	23 (6.0)	55 (14.4)
Do you maintain social distance (or home quarantine)?	229 (59.9)	66 (17.3)	87 (22.8)
Do you use tissues or hanker chips during coughing/sneezing?	228 (59.7)	89 (23.3)	65 (17.0)
Do you eat healthy food or maintain a healthy lifestyle focusing on outbreaks?	255 (66.8)	40 (10.5)	87 (22.8)
Do you avoid public transports (like buses, trains, planes, etc.)?	169 (44.2)	123 (32.2)	90 (23.6)
Do you avoid handshaking, hugging when you meet with your friends?	223 (58.4)	83 (21.7)	76 (19.9)

** This question had "Not possible for work" instead of "Maybe" in its three options. n- Number of respondents, %- Percentage of respondents, N- Total number of respondents

Fear and its factors

On average, participants had a 35.8% fear score (MS ± SD = 10.03 ± 4.77 out of 28). 77.7% (297) participants had less fear, 20.2% (77) had moderate fear, and only 2.1% (8) participants had high fear. And 77.7% (297) participants had very less fear (less than 50% fear score) while only 22.3% (85) participants had high to moderate fear (equal or more than 50% fear score). The distribution of responses of all fear-related questions was presented in (Table 3). Mann-Whitney and Kruskal-Wallis tests unfolded that fear score among participants varies with only gender ($P < 0.001$). It

was reported that females had a significantly high fear score than males (Table 4). Multiple logistic regression analysis detected gender, knowledge, attitude, practice, as significant factors that can predict whether an individual would have very little fear rather than high to moderate fear. It was found that male participants were 89% more likely to have very less fear than female participants (AOR = 1.89, 95% CI = 1.05-3.40, $P < 0.05$) (Table 5).

Table 3

Fear related to covid-19 among respondents (N= 382) (Distribution of responses).

Fear Questions	Strongly Disagree n (%)	Disagree n (%)	Neutral n (%)	Agree n (%)	Strongly Agree n (%)
I am most afraid of coronavirus-19.	43 (11.3)	96 (25.1)	124 (32.5)	90 (23.6)	29 (7.6)
It makes me uncomfortable to think about coronavirus-19.	35 (9.2)	118 (30.9)	93 (24.3)	109 (28.5)	27 (7.1)
My hands become clammy when I think about coronavirus-19.	119 (31.2)	216 (56.5)	29 (7.6)	15 (3.9)	3 (0.8)
I am afraid of losing my life because of coronavirus-19.	47 (12.3)	134 (35.1)	100 (26.2)	81 (21.2)	20 (5.2)
When watching news and stories about coronavirus-19 on social media, I become nervous or anxious.	45 (11.8)	117 (30.6)	103 (27.0)	86 (22.5)	31 (8.1)
I cannot sleep because I'm worried about getting coronavirus-19.	132 (34.6)	204 (53.4)	32 (8.4)	13 (3.4)	1 (0.3)
My heart races or palpitates when I think about getting coronavirus-19.	127 (33.2)	180 (47.1)	49 (12.8)	24 (6.3)	2 (0.5)

n- Number of respondents, %- Percentage of respondents, N- Total number of respondents

Relationship among knowledge, attitude, practice, and fear

Spearman rank-order correlation tests explored that knowledge score is significantly correlated with attitude score ($P < 0.01$) although it was insignificantly correlated with other scores (practice and fear score). However, attitude score was positively correlated with both practice score ($P < 0.001$) and fear score ($P < 0.001$). Along with attitude score, practice score and fear score was positively correlated with each

other (P < 0.01) (Table 6). Multiple logistic regression analysis uncovered that the participants who had more accurate knowledge were 2.34 times more likely to have a more positive attitude (AOR = 2.34, 95% CI = 1.23-4.47, P < 0.01) and 2.17 times more likely to have very little fear (AOR = 2.17, 95% CI = 1.10-4.26, P < 0.05) than those who had less accurate knowledge; Participants who had a more positive attitude were 4 times more likely to have more frequent practice than those who had less positive attitude (AOR = 4.00, 95% CI = 2.44-6.56, P < 0.001). Moreover, the participants who had very less fear were 56% less likely to have more positive attitude (AOR = 0.44, 95% CI = 0.23-0.84, P < 0.01) and at the same time 53% less likely to have more frequent practice (AOR = 0.47, 95% CI = 0.26-0.84, P < 0.01) than those who had high to moderate fear (Table 5).

Table 4

Association of socio-demographic characteristics with knowledge, attitude, fear, and Practice score.

**Socio-demographic characteristics (n)**	**Knowledge**		**Attitude**		**Fear**		**Practice**	
	Mean Rank	U/H test P-value	Mean Rank	U/H test P-value	Mean Rank	U/H test P-value	Mean Rank	U/H test P-value
**Gender***								
Male (285)	195.32	0.225	186.58	0.130	176.37	**<0.001**	175.86	**<0.001**
Female (97)	180.26		205.95		235.96		237.46	
**Education***								
HSC (104)	183.24	0.349	200.50	0.323	204.98	0.144	206.07	0.112
BSc/MSc/Above(278)	194.59		188.13		186.46		186.05	
**Occupation***								
Student and self-employed (152)	197.62		177.89		181.38		180.61	

Student only (230)	187.45	0.357	200.49	**0.047**	198.19	0.144	198.70	0.115
**Family type***								
Nuclear (293)	188.11	0.225	187.76	0.224	188.37	0.313	194.41	0.347
Extended (89)	202.65		203.80		201.81		181.93	
**Income****								
<20,000 BDT (222) **(a)**	187.35	0.647	179.91	**0.05**	194.48	0.622	173.86	**0.001**
20,000-40,000 BDT (126) **(b)**	196.30		207.26		183.97		212.09	
>40,000 BDT (34) **(c)**	200.82		208.76		199.97		230.37	
**Location****								
Village (142) **(d)**	182.30	0.360	191.30	0.425	183.32	0.484	166.96	**0.004**
Sub-district town (39) **(e)**	210.58		197.91		182.69		189.85	
District town (97) **(f)**	187.76		203.31		204.63		217.15	
Divisional district town (104)	200.39		178.35		193.73		201.70	

BDT - Bangladesh Taka (Currency symbol of Bangladesh taka), HSC = Higher Secondary School Certificate, BSc- Bachelor of Science, MSc- Masters of Science, p- Significance value, *U = Mann-Whitney U, **H = Kruskal-Wallis H

[**(a)** vs **(b)** and **(a)** vs **(c)** were significant at 0.005 and 0.015 level respectively in practice (Bonferroni corrected)**; (a)** vs **(b)** was significant at 0.024 level in attitude (without Bonferroni correction)**; (d)** vs **(e)** was significant at 0.003 in practice (Bonferroni corrected)**; (d)** vs **(f)** was significant at 0.014 level in practice (without Bonferroni correction)]

Table 5

Results of multiple logistic regression on factors associated with knowledge, attitude, practice, and fear. (Adjusted model)

**Variables (D)**	**More Accurate knowledge**		**More Positive attitude**		**More frequent practice**		**Very little fear**	
	n (%)	AOR (95% CI)	n (%)	AOR (95% CI)	n (%)	AOR (95% CI)	n (%)	AOR (95% CI)
**Gender**								
Male (285)	243 (85.3)	1.14 (0.56-2.32)	182 (63.9)	1.22 (0.67-2.20)	140 (49.1)	**0.51* (0.29-0.89)**	232 (81.4)	**1.89* (1.05-3.40)**
Female (97)	81 (83.5)	ref	70 (72.2)	ref	69 (71.1)	ref	65 (67.0)	ref
**Education**								
HSC (104)	91 (87.5)	ref	74 (71.1)	ref	62 (59.6)	ref	79 (76.0)	ref
BSc/MSc/Above (278)	233 (83.8)	0.73 (0.36-1.49)	178 (64.0)	0.83 (0.47-1.45)	147 (52.9)	0.94 (0.55-1.62)	218 (78.4)	0.96 (0.53-1.74)
**Occupation**								
Student and self-employed (152)	130 (85.5)	ref	93 (61.2)	ref	74 (48.7)	ref	124 (81.6)	ref
Student only (230)	194 (84.3)	0.90 (0.47-1.73)	159 (69.1)	1.09 (0.65-1.84)	135 (58.7)	1.20 (0.73-1.99)	173 (75.2)	0.87 (0.48-1.58)
**Family type**								
Nuclear (293)	246 (84.0)	ref	189 (64.5)	ref	163 (55.6)	ref	230 (78.5)	ref
Extended (89)	78 (87.6)	1.45 (0.70-3.01)	63 (70.8)	1.28 (0.73-2.25)	46 (51.7)	0.86 (0.50-1.46)	67 (75.3)	0.73 (0.40-1.33)
**Income**								
<20,000 BDT (222)	184	0.89	135	**0.33***	103	0.63	177	0.53

	(82.9)	(0.27-3.0)	(60.8)	**(0.11-0.94)**	(46.4)	(0.26-1.55)	(79.7)	(0.19-1.40)
20,000-40,000 BDT (126)	110 (87.3)	1.09 (0.31-3.78)	89 (70.6)	0.41 (0.14-1.19)	81 (64.3)	1.06 (0.42-2.65)	93 (73.8)	0.50 (0.19-1.33)
>40,000 BDT (34)	30 (88.2)	ref	28 (82.4)	ref	25 (73.5)	ref	27 (79.4)	ref
**Location**								
Village (142)	113 (79.5)	ref	92 (64.8)	ref	63 (44.4)	ref	115 (81.0)	ref
Sub-district town (39)	37 (94.9)	**4.85* (1.07-22.05)**	26 (66.7)	0.844 (0.37-1.93)	19 (48.7)	0.98 (0.44-2.19)	31 (79.5)	0.92 (035-2.40)
District town (97)	83 (85.6)	1.46 (0.69-3.08)	71 (73.2)	0.99 (0.53-1.89)	63 (64.9)	**2.01* (1.09-3.65)**	72 (74.2)	0.78 (0.39-1.54)
Divisional district town (104)	91 (87.5)	2.03 (0.93-4.40)	63 (60.6)	**0.50* (0.27-0.93)**	64 (61.5)	**2.04* (1.12-3.73)**	79 (76.0)	0.66 (0.33-1.31)
**Knowledge**								
More accurate knowledge (324)	-	-	222 (68.5)	**2.34** (1.23-4.47)**	179 (55.2)	0.94 (0.49-1.80)	257 (79.3)	**2.17* (1.10-4.26)**
Less accurate knowledge (58)	-	-	30 (51.7)	ref	30 (51.7)	ref	40 (69.0)	ref
**Attitude**								
More positive attitude (252)	222 (88.1)	**2.39** (1.26-4.54)**	-	-	168 (66.7)	**4.00*** (2.44-6.56)**	183 (72.6)	**0.43** (0.22-0.82)**
Less positive attitude (130)	102 (78.5)	ref	-	-	41 (31.5)	ref	114 (87.7)	ref
**Practice**								
More frequent practice (209)	179 (85.6)	0.98 (0.51-1.88)	168 (80.4)	**4.01*** (2.44-6.55)**	-	-	146 (69.9)	**0.46** (0.26-0.83)**
Less frequent practice (173)	145 (83.8)	ref	84 (48.6)	ref	-	-	151 (87.3)	ref
**Fear**								
High to moderate fear (85)	67 (78.8)	ref	69 (81.1)	ref	63 (74.1)	ref	-	-
Very little fear (297)	257 (86.5)	**2.25* (1.13-4.47)**	183 (61.6)	**0.44** (0.23-0.84)**	146 (49.2)	**0.47** (0.26-0.84)**	-	-
**Total**	324 (84.8%)		252 (66.0%)		209 (54.7%)		297 (77.7%)	

D = Number of respondents in each category of independent variables, n = Number of respondents in category of dependent variables, BSc- Bachelor of Science, MSc- Masters of Science, AOR = Adjusted Odd ratio, Reference category = ref,
*P ≤ 0.05, **P ≤ 0.01, ***P ≤ 0.001, High to moderate fear = equal or more than 50% fear score, Very less fear = less than 50% fear score.

Table 6

Results of spearman's rank-order correlation among knowledge, attitude, practice, and fear score.

	1	2	3	4
**1**. Knowledge	1			
**2**. Attitude	0.12[a] 0.015[b]	1		
**3**. Practice	0.05[a] 0.369[b]	0.36[a] < 0.001[b]	1	
**4**. Fear	-0.05[a] 0.280[b]	0.24[a] < 0.001[b]	0.15[a] 0.005[b]	1

a = Correlation coefficient, b = Significance level (Two tailed)

Probable reasons explaining why students are detached from maintaining CPMs

When asked to observe participants' thoughts as to whether they are maintaining safety rules for Covid-19, 27% (103) participants assured that they are maintaining safety rules completely while 73% (279) reported "No" and mentioned their possible reasons why they were detached from maintaining safety rules properly. Among those who responded "No", 56.3% participants mentioned they cannot maintain a social distance because they need to go out for many reasons and need to use public transports where social distance maintaining is not possible while 44.8% participants mentioned they need to go market for daily groceries or many things where they cannot maintain social distance. 28.3% of the participants think of themselves as social butterflies and meet with many friends while they do not maintain social distance. 22.2% of the participants reported that they can't wash their hands regularly while 32.0% mentioned that very few times a day, they wash their hands as recommended by WHO. The further analysis explored that among participants who mentioned they wash their hands regularly, 22.1% do not wash their hands maintaining rules recommended by WHO while 77.8% wash their hands regularly as recommended by WHO and among those who mentioned they do not wash their hands regularly, 66.1% maintain rules recommended by WHO when they wash their hands. In addition, 37.6% of participants don't use hand sanitizers when staying outside the home and 23.3% of participants have bad habits of touching eyes, nose,

mouth, frequently. 21.1% of the participants reported that they have not seen anyone from their relatives or relatives of relatives or neighbors got infected by a coronavirus and that is why they do not give importance to maintaining safety rules of Covid-19. (Fig 2) depicted all the other reasons graphically with the frequency of responses for each reason and (Table 7) tabulated all the supportive information.

Fig 2 Graphical representation of reasons behind failure to maintain safety guidelines of covid-19. (Table 7) tabulated all the supportive information.

Table 7

Possible reasons why people cannot maintain safety rules of Covid-19 completely.

▪ Beliefs that I will not be infected by the coronavirus. **(C01)**
▪ Things related to corona seem to me as media-created rumors. **(C02)**
▪ No one having been identified as corona virus-positive yet from my relatives or relatives of relatives or neighbors makes maintaining safety rules less important to me. **(C03)**
▪ I am less panicked about coronavirus. **(C04)**
▪ I have no clear conception about safety rules for Covid-19. **(C05)**
▪ It is not always possible for me to wash hands coming from outsides. **(C06)**
▪ Very few times in a day, hands are washed up to the elbow for (20-30) min as recommended by WHO. **(C07)**
▪ I do not use hand sanitizer when staying outside. **(C08)**
▪ Trying but I can't give up the bad habit of touching eyes, face, nose frequently. **(C09)**
▪ I am to go to the market often for daily groceries or other things where maintaining social distance is not possible. **(C10)**
▪ I am to go out for many reasons and use public transports where maintaining social distance is not possible. **(C11)**
▪ I am a social butterfly and hang out with friends very often while social distance is not maintained. **(C12)**
▪ I don't maintain social distance thinking about the benefit of maintaining social distance when people around me don't maintain social distance. **(C13)**
▪ I don't use medical masks because of having any confidence in a normal medical mask (5 BDT) as well as no money for buying expensive masks**. (C14)**
▪ When wearing a mask, I feel very uneasy and it's very hard to stay with. **(C15)**
▪ I don't use a mask because it looks ugly. **(C16)**
▪ I don't know any reason. **(C17)**

BDT-Bangladesh Taka (Currency symbol of Bangladeshi taka)

Discussion

To fulfill the goals of this present study, several statistical analyses had been conducted and their outcomes had been presented sequentially in the result section. In this section, these outcomes have been discussed collaboratively with other previously published studies.

Knowledge level of Participants

The findings showed that participants' overall correct answer rate was 89.6%, indicating participants had appreciable Knowledge on Covid-19. This outcome was consistent with some previous studies conducted last year (2020) in Bangladesh (80.8%) (18), China (90%) (19), Malaysia (80.5%) (20), Saudi Arabia (81.6%) (21), Iran (90% & 85%) (22), Nepal (60% to 98.7%) (23). Moreover, 84.8% of participants had more accurate knowledge which was larger compared to that reported by some previous Bangladeshi investigations (7–9,11,13), indicating knowledge level has elevated among Bangladeshis.

Our study reported that knowledge scores do not significantly vary with gender, education, occupation, location, income, family type, which was inconsistent with some studies (7–9,11,13,19,24).

Attitude level of Participants

Approximately 66% of participants had a more positive attitude towards Covid-19, which was commensurate with an article (11). But when compared to the other two Bangladeshi articles, it seems attitude level among students has alleviated to some extent (8,9). Compared to overseas investigations, our finding was consistent with a study from Pakistan (65.4%) (12), while inconsistent with studies from Saudi Arabia (95%) (25), China (73.8%) (10). Our findings suggested that Bangladeshi students might have lost their confidence towards controlling the rapid escalation of coronavirus as only 35.3% of participants took it confidently that Bangladesh can win the battle against Covid-19 and concomitantly, only 44.2% of participants were confident that Covid-19 will finally be controlled. Last year, Rabbani et al., (2020) reported that 55.3% of their participants were confident about Bangladesh would win the battle against Covid-19 which was also consistent with Kundu et al., n.d. (2020), and 68.5% were confident that Covid-19 would finally be controlled which was somewhat lower compared to Banik et al., (2020) (9,13,26). On the other hand, a Malaysian, Chinese, Saudi Arabian, Indonesian, Nepali article published last year (2020) reported that they respectively obtained 83.1%, 90.8%, 94%, 94.3%, and 71.5% positive responses regarding whether Covid-19 would successfully be controlled, and 95.9%, 97.1%, 97%, 95.5%, and 80% positive response regarding whether their country would be able to win the battle against Covid-19 (19–

21,24,27).On the whole, it is obtrusive that attitude level among students were noticeably subsided. The reason behind obtaining such ominous outcomes might be the long duration of uncertainties which has been appeared due to emerging of new variants of SARS-COV-2 with the function of time.

Furthermore, our study explored that attitude levels significantly vary with occupation, and family income of the participants, not with other socio-demographic variables included in our scrutiny, which was somewhat inconsistent with several studies. (7,9,11).

Fear level of Participants

On average, participants had a 35.8% fear score which was lower compared to Hossain MA, et al., (2020) (28). Besides, only 2.1% of participants had very high fear while 77.7% had very less fear. These outcomes indicate that Bangladeshi students are no longer panicked. The fear score was significantly associated with only gender of the participants. Females had significantly high fear than males. Hossain MA, et al., (2020) reported that the fear score significantly differs with gender, education, and geography, when female, urban-dwelling, and higher educated participants had high fear, which was partially similar to our findings (28).

Practice level of Participants

On average, participants had a 74.2% practice score and 55% participants had more frequent practice, which was consistent with some studies from Bangladesh (7,9,11,26). So, our study indicates that the preventive practice level remains similar to what was in last year. However, compared to a study from Bangladesh (24%) (13) and from Pakistan (36.5%) (12), our participants had more frequent practice but seems to have much lower frequent practice when compared to the studies from Saudi Arab (81% & 81.9%) (14,25) and South Korea (87.9%) (10). Indeed, our study found that more frequent practice is greatly influenced by gender, family income and location. However, the more frequent practice could also be influenced by other socio-demographic factors such as education, occupation, age, etc. (8,9,11,13).

Relationship among knowledge, attitude, practice, and fear

The finding showed knowledge has an indirect effect on preventive practice mediated by attitude as knowledge has a positive impact on attitude and concomitantly, an attitude has a positive impact on preventive practice, indicating the more knowledge on Covid-19, the more attitude towards it, and the more frequent practice towards its prevention. Similar outcomes were reported in some other studies (8,13,15,25,29,30). Moreover, our study uncovered that knowledge has a significant inverse effect on fear, indicating the more accurate knowledge, the less fear. This outcome was parallel with some other studies (18,25,28,31). Our study

also revealed that like attitude, fear has also a positive impact on preventive practice, indicating the more fear, the more preventive practice. A similar outcome was reported by Hossain MA, et al,.(2020) & W/Mariam TG, et al,.(2020) (28,32). Ostensibly, this outcome could seem controversial but it represents the significant influence of fear on changing someone's behavior or attitude towards preventive practice. Our study also substantiates the immediate statement reporting that fear has a positive and mysteriously similar impact on attitude as was on preventive practice. However, we cannot expect high fear among the populace to have more practice because fear has some shortcomings too. For instance, Covid-19 fear is significantly associated with anxiety and psychological distress, which could in turn trigger stress and mental illness (31). Besides, there is an instance of suicidal death due to the fear of Covid-19 in Bangladesh (28,33). (Fig 3) depicted all the factors and their impact on preventive practice.

Fig 3 Diagram showing the direct and indirect impact of knowledge, attitude, fear, and socio-demographic factors (Ellipse) on preventive practice.

~~In addition, Our study reported that the majority of the participants thought they cannot adhere to preventive measures because of not maintaining social distance properly, followed by the reluctance in hand washing as recommended by WHO. We had no sufficient data to support these reasons.~~

Limitations, Strength, and Recommendation

Our study had several limitations. First, our study sample could not reflect all students throughout the country. Second, our study could not include students of all ages. Third, the study sample size was comparatively small. Fourth, our study followed a web-based cross-sectional study that excluded underprivileged students especially those who were not accessible to internet-based facilities. Five, we had to conduct comparatively less sensitive statistical analysis as our data were not normally distributed. As to the strength of our study, to the best of our knowledge, this is the first study that attempted to find out possible reasons why students are deflected from maintaining preventive measures. Future investigations should follow both community-based and web-based cross-sectional studies, collecting data from all sectors, all divisions, attempting to find out the most probable reasons behind failure to maintain all CPMs in a more structured way.

Conclusion

Our study attempted to bring up the present knowledge, attitude, practice, and fear level of Bangladeshi students. According to our study, students had appreciable knowledge and very little fear but disappointedly had average attitude and practice towards CPMs. Besides, students had a lack of confidence on Bangladesh would win the battle against Covid-19. Therefore, it is time for public health policymakers to

be more focused to scale up students' confidence and attitude towards Covid-19. To do so, they must be more dogmatic to develop rules and regulations and more austere in implementing and maintaining those to combat any new onslaught of the deadly coronavirus. Besides, providing public health educations, arranging viable public health campaigns, promoting accurate information regarding Covid-19, counseling students as well as general people with regards to preventive practices, must be continued till the end of this unprecedented condition.

Supporting Information

S 1 File. Questionnaire and Coding information. (DOCX)

S 2 File. Covid-19 Raw Data. (XLSX)

Acknowledgments

The authors acknowledge all the participants who participated in the study and all the volunteers who helped in data collection. The authors are also grateful to prof. Dr. Md. Golam Sadik & Dr. Md. Abdul Rafi for their support in the overall improvement of the manuscript.

Author contributions

Conceptualization: Tahsin Ahmed Rupok, Sunandan Dey

Data curation: Tahsin Ahmed Rupok, Rashni Agarwala, Md. Nurnobi Islam, Sunandan Dey, Bayezid Bostami.

Formal Analysis: Tahsin Ahmed Rupok, Rashni Agarwala.

Investigation: Tahsin Ahmed Rupok, Rashni Agarwala, Md. Nurnobi Islam, Sunandan Dey.

Methodology: Tahsin Ahmed Rupok, Bayezid Bostami.

Project Administration: Tahsin Ahmed Rupok, Sunandan Dey.

Supervision: Tahsin Ahmed Rupok, Sunandan Dey.

Writing – original draft: Tahsin Ahmed Rupok.

Writing – review & editing: Tahsin Ahmed Rupok, Md. Nurnobi Islam, Sunandan Dey, Rashni Agarwala, Bayezid Bostami.

Reference

1. Correspondent S. Country logs highest Covid cases in a day. Daily Star [Internet]. 2021 Jul 1; Available from: https://www.thedailystar.net/news/bangladesh/news/country-logs-highest-covid-cases-day-2121453
2. Correspondent S. 112 more die, 7,666 get Covid. Daily Star [Internet]. 2021

Jun 30; Available from: https://www.thedailystar.net/bangladesh/news/112-more-die-7666-get-covid-2120757

3. SARS-CoV-2 Delta variant [Internet]. Available from: https://en.wikipedia.org/wiki/SARS-CoV-2_Delta_variant

4. Bangladesh could face severe Covid-19 surge by June-end if Indian variant spreads: experts. Daily Star [Internet]. 2021 May 25; Available from: https://www.thedailystar.net/coronavirus-deadly-new-threat/news/bangladesh-could-face-severe-covid-19-surge-june-end-if-indian-variant-spreads-experts-2098725

5. Majumdar BA. How do we tackle this new onslaught of the coronavirus? Daily Star [Internet]. 2021 Jun 28; Available from: https://www.thedailystar.net/opinion/news/how-do-we-tackle-new-onslaught-the-coronavirus-2119313

6. Correspondent S. Lockdown now from Thursday. Daily Star [Internet]. 2021 Jun 27; Available from: https://www.thedailystar.net/frontpage/news/lockdown-now-thursday-2119017

7. Paul A, Sikdar D, Hossain MM, Amin MR, Deeba F, Mahanta J, et al. Knowledge, attitudes, and practices toward the novel coronavirus among Bangladeshis: Implications for mitigation measures. PLoS One [Internet]. 2020;15(9 September 2020):1–18. Available from: http://dx.doi.org/10.1371/journal.pone.0238492

8. Hossain MB, Alam MZ, Islam MS, Sultan S, Faysal MM, Rima S, et al. Do knowledge and attitudes matter for preventive behavioral practices toward the COVID-19? A cross-sectional online survey among the adult population in Bangladesh. Heliyon. 2020;6(12). Available from: https://doi.org/10.1016/j.heliyon.2020.e05799

9. Banik R, Rahman M, Sikder M, Rahman QM, Pranta MUR. Investigating knowledge, attitudes, and practices related to COVID-19 outbreak among Bangladeshi young adults: A web-based cross-sectional analysis. J public Heal [Internet]. 2020 [cited 2021 Mar 31]; Available from: https://doi.org/10.1007/s10389-020-01432-7

10. Peng Y, Pei C, Zheng Y, Wang J, Zhang K, Zheng Z, et al. A cross-sectional survey of knowledge , attitude and practice associated with COVID-19 among undergraduate students in China. BMC Public Health [Internet]. 2020; Available from: https://doi.org/10.1186/s12889-020-09392-z

11. Ferdous MZ, Islam MS, Sikder MT, Mosaddek ASM, Zegarra-Valdivia JA, Gozal D. Knowledge, attitude, and practice regarding COVID-19 outbreak in Bangladesh: An onlinebased cross-sectional study. PLoS One [Internet]. 2020;15(10 October):1–17. Available from: http://dx.doi.org/10.1371/journal.pone.0239254

12. Salman M, Ul Z, Noman M, Abbas H, Khalid Z. Knowledge , attitude and preventive practices related to COVID ‑ 19 : a cross ‑ sectional study in two Pakistani university populations. Drugs Ther Perspect [Internet]. 2020;36(7):319–25. Available from: https://doi.org/10.1007/s40267-020-00737-7

13. Rabbani MG, Akter O, Hasan MZ, Samad N, Mahmood SS, Joarder T. Knowledge, Attitude and Practice towards COVID-19 among people in Bangladesh during the pandemic: a cross-sectional study. medRxiv [preprint] [Internet]. 2020; Available from: https://doi.org/10.1101/2020.09.22.20198275

14. Alhazmi A, Hamed M, Ali M, Mohieldin A, Aziz F, Osman OB, et al. Knowledge , attitudes and practices among people in Saudi Arabia regarding COVID-19 : A cross-sectional study. J Public health Res [Internet]. 2020;9(3):345–53. Available from: https://www.ncbi.nlm.nih.gov/pmc/articles/PMC7520855/

15. Lee M, Kang BA, You M. Knowledge, attitudes, and practices (KAP) toward COVID-19: a cross-sectional study in South Korea. BMC Public Health. 2021;21(1):1–10. Available from: https://doi.org/10.1186/s12889-021-10285-y

16. Tayeb T. Bangladesh's Covid lockdown conundrum. Daily Star [Internet]. 2021 Apr 18; Available from: https://www.thedailystar.net/opinion/closer-look/news/bangladeshs-covid-lockdown-conundrum-2079065?amp

17. Ahorsu DK, Lin C, Imani V, Saffari M, Griffiths MD, Pakpour AH. The Fear of COVID-19 Scale : Development and Initial Validation. Int J Ment Health Addict [Internet]. 2020; Available from: https://doi.org/10.1007/s11469-020-00270-8

18. Ali M, Uddin Z, Banik PC, Hegazy F, Zaman S, Ambia ASM, et al. Knowledge, attitude, practice and fear of COVID-19: A cross-cultural study. medRxiv [preprint]. 2020;1–27. Available from: https://doi.org/10.1101/2020.05.26.20113233

19. Zhong B, Luo W, Li H, Zhang Q, Liu X, Li W, et al. Knowledge , attitudes ,

and practices towards COVID-19 among Chinese residents during the rapid rise period of the COVID-19 outbreak : a quick online cross-sectional survey. Int J Biol Sci. 2020;16(10). Available from: https://doi.org/10.7150/ijbs.45221

20. Azlan AA, Hamzah MR, Jen T, Id S, Hadi S, Id A. Public knowledge , attitudes and practices towards COVID-19 : A cross-sectional study in. PLoS One [Internet]. 2020;15(5):1–15. Available from: http://dx.doi.org/10.1371/journal.pone.0233668

21. Al-Hanawi MK, Angawi K, Alshareef N, Qattan AMN, Helmy HZ, Abudawood Y, et al. Knowledge, Attitude and Practice Toward COVID-19 Among the Public in the Kingdom of Saudi Arabia: A Cross-Sectional Study. Front Public Heal. 2020;8(May):1–10. Available from: https://doi.org/10.3389/fpubh.2020.00217

22. Erfani A, Shahriarirad R, Ranjbar K, Mirahmadizadeh A, Moghadami M. Knowledge, attitude and practice toward the novel coronavirus (COVID-19) outbreak- A population-based survey in Iran. Bull World Health Organ [Internet]. 2020; Available from: http://dx.doi.org/10.2471/BLT.20.256651

23. Hussain A, Garima T, Singh BM, Ram R, Tripti RP. Knowledge, attitudes, and practices towards COVID-19 among Nepalese Residents: A quick online cross-sectional survey. Asian J Med Sci. 2020;11(3):6–11. Available from: https://doi.org/10.3126/ajms.v11i3.28485

24. Paudel S, Shrestha P, Karmacharya I, Pathak OK. Knowledge, attitude, and practices (KAP) towards COVID-19 among Nepalese residents during the COVID-19 outbreak: An online cross-sectional study. 2020; Available from: https://doi.org/10.21203/rs.3.rs-31044/v1

25. Alahdal H, Basingab F, Alotaibi R. An analytical study on the awareness, attitude and practice during the COVID-19 pandemic in Riyadh, Saudi Arabia. J Infect Public Health [Internet]. 2020;13(10):1446–52. Available from: https://doi.org/10.1016/j.jiph.2020.06.015

26. Kundu S, Sayeed A, Banna MH Al, Begum MR, Kormoker T, Brazendale K, et al. Knowledge, attitudes, and practices towards measures for prevention of the spread of COVID-19: An online cross-sectional survey among Bangladeshi residents. PsyArxiv [Preprints] [Internet]. :1–22. Available from: https://doi.org/10.31234/osf.io/cxp8n

27. Muslih M, Susanti HD, Rias YA. Knowledge , Attitude , and Practice of Indonesian Residents toward COVID-19 : A Cross-Sectional Survey. Int J

Environ Res Public Health [Internet]. 2021; Available from: https://doi.org/10.3390/ijerph18094473

28. Hossain MA, Hossain KMA, Walton LM, Uddin Z, Haque MO, Kabir MF, et al. Knowledge, attitudes, and fear of COVID-19 during the rapid rise period in Bangladesh. medRxiv [Internet]. 2020 [cited 2021 Mar 31]; Available from: https://journals.plos.org/plosone/article?id=10.1371/journal.pone.0239646

29. Lau LL, Hung N, Go DJ, Ferma J, Choi M, Dodd W, et al. Knowledge, attitudes and practices of COVID-19 among income-poor households in the Philippines: A cross-sectional study. J Glob Health. 2020;10(1). Available from: https://doi.org/10.7189/JOGH.10.011007

30. Gao H, Hu R, Yin L, Yuan X, Tang H, Luo L, et al. Knowledge, attitudes and practices of the Chinese public with respect to coronavirus disease (COVID-19): An online cross-sectional survey. BMC Public Health. 2020;1–8. Available from: https://doi.org/10.21203/rs.3.rs-40292/v1

31. Saravanan C, Mahmoud I, Elshami W, Taha MH. Knowledge, Anxiety, Fear, and Psychological Distress About COVID-19 Among University Students in the United Arab Emirates. Front Psychiatry. 2020;11(October):1–10. Available from: https://doi.org/10.3389/fpsyt.2020.582189

32. W/Mariam TG, Kassie BA, Asratie MH, Abate AT. The Effects of Fear and Knowledge of COVID-19 on Preventive Practice Among Pregnant Women Who Attend Antenatal Care in Northwest Ethiopia, 2020: Institution-Based Cross-Sectional Study. Int J Womens Health. 2021;Volume 13:95–100. Available from: https://doi.org/10.2147/ijwh.s286088

33. Mamun MA, Gri MD. First COVID-19 suicide case in Bangladesh due to fear of COVID-19 and xenophobia : Possible suicide prevention strategies. Asian J Psychiatr. 2020;51(April):2–3. Available from: https://doi.org/10.1016/j.ajp.2020.102073

Dear editor,

Here I am writing our responses to each points raised by the reviewers.

Responses to the queries of Reviewer #1:

Thanks for your valuable comments. According to your suggestion, all the authors critically revised the manuscript again. Besides, we requested another expert (name mentioned on the Acknowledgment section) to revise our manuscript. Hopefully, his contribution would add significant value to this manuscript. (All the changes have been marked on the “Revised Manuscript with Track Changes”)

Responses to the queries of Reviewer #2:

Thanks for your valuable suggestion. I provided the approval letter (scanned copy of the original letter) as a supplementary file for strengthening the credibility of ethical issue of the study.
