# Supplementary material for: Knowledge, attitude, practice, and fear level of Bangladeshi students toward Covid-19 after a year of the pandemic situation: A web-based cross-sectional study
Source: PLoS One. 2023 Feb 27;18(2):e0282282. doi: 10.1371/journal.pone.0282282 (PMC9970072; doi:10.1371/journal.pone.0282282)
Supplement: Attachment — Submitted filename: Response to Reviewers.docx [file pone.0282282.s005.docx]

Dear editor,

Here I am writing our responses to each points raised by the reviewers.

Responses to the queries of Reviewer #2:

Title:

Better to omit the word ‘Present’ from the title. It is suggested to keep the title as ‘Knowledge, attitude, practice, and fear level of Bangladeshi people towards covid-19 after a year of the pandemic situation: a web-based cross-sectional study’

Author: I made changes as you suggested. Please vide the title page.

This study has been conducted mainly among the students (out of 402 samples, 382 are students) and does not necessarily represent Bangladeshi people as a whole, therefore, it is suggested to make changes in the title of the manuscript.

Author: I made changes as you suggested. Please vide the title page. I excluded all the participants who were not student.

Abstract:

Age of the study participants ranged from 16-30 meaning that all were not adults, so the authors are requested to make correction in the abstract and report the percentage of the participants <18 years.

Author: I made correction as you suggested. Please vide (line 38). I could not report exact percentage of the participants <18 as in our questionnaire, under the question “what is your age category?” there was options like below 16 or 16-30 or more than 30 years old.

Please report the exact percentage, not like more than 90%.

Author: I made correction as you suggested. Please vide (line 38).

Background:

Overall, the background section needs to be re-written. It does not well justify the study.

Please do not mention individual’s name that can be identified easily and give reference of the statement. If it is personal communication, follow the reference style for that as well (lines 59-65)

Author: I made correction as you suggested. (Lines 60-65)

Please mention the exact year instead of ‘this year’ (Line 76)

Author: I made change as you suggested. (Line 71)

The authors should mention ‘The Government of Bangladesh (GoB)’ throughout the paper, instead of ‘Bangladesh’s government’

Author: I made change as you suggested (line 73)

Please mention the exact year instead of ‘last year’ (line 82)

Author: I made correction as you suggested (Line 77)

Please give reference for the statement (lines 81-86) and follow the reference style instructed in author guideline

Author: I did give reference. (Line 80)

I don’t see any figure or diagram in the document (missing!)

Author: Figures or diagrams were uploaded separately with name like “Fig 1, or Fig 2”

Methodology:

It is not clear how the participants were approached and selected.

Author: I reported how the participants were approached and selected. (Lines 122-126)

It seems that the study was conducted among the Bangladeshi student. Please make it clear, make correction accordingly throughout the paper.

Author: I did perform statistical analysis on the participants as you suggested and made correction throughout the paper accordingly.

Please write ‘participants’ or ‘respondents’ instead of study ‘subjects’

Author: I made correction as you said (line 127).

Income is not sufficient to determine social status and classify as ‘lower class, middle class and high class’. It is suggested to keep it simple as ‘income’ and categorize the income using range.

Author: I made correction as you suggested (136)

Ethical consideration:

The authors should mention how they have taken assents from the participants of <18 years

Author: Assents from the participants of <18 years were taken thorough online approaches as were taken from other participants. This study included only those who had online accesses.

Data management and analysis:

The author should mention why they performed different statistical test and what they found from each test (i.e., how was the distribution of data under the variable(s)?)

Author: I mentioned the purposes of the different statistical tests (line 189-196), reporting the outcomes of those tests in result section. I mentioned the distribution of data under the variables (line 196-203)

What was/were the outcome(s) of interest in regression model? what independent variables were taken into consideration? And level of significance considered during keeping the variables in final model.

Author: I reported the outcomes of interest and independent variables taken in regression model (line 193—96). I mentioned level of significance for all the analysis in together (line 203).

The authors performed MLR; why didn’t they report adjusted OR?

Author: I made correction throughout the paper as you suggested.

Results:

Overall, the results are not well interpreted. The authors have been suggested organize, present and interpret the results in scientific manner. Examples are numerous:

Please mention the equivalent grades instead of ‘honors and masters’ (line 207)

Author: I made correction throughout the paper as you suggested (line 209).

It seems that the participants were the students, not from other occupations. The authors have been suggested to rewrite and interpret the result section accordingly.

Author: I excluded participants from occupations other than student, performed analysis, and interpreted the result section accordingly.

‘Participants’ Overall correct answer rate was 89.6% [Mean Score (MS) ± Standard 218 Deviation (SD) = 10.75±1.20 out of 12] while the correct answer rate was 65% to 219 100%, indicating participants had appreciable knowledge on covid-19.’ Interpretation of the results is confusing. Please rephrase.

Author: I made correction as you suggested (line 220-222).

Responses to some queries of Reviewer #1:

Majority of the queries and suggestions were similar with Reviewer #2. Here I am mentioning those which were somewhat exceptional.

The cut-off point for categorization of knowledge, attitude, practice, and fear is not clear. For example, why 81% was selected as a cut-off point for practice? It should be made clear in the methods section.

Author: I made correction and mentioned in methods section (line 155-167).

Why location was categorized in this way? What is the reason behind taking ‘Dhaka’ as a different location?

Author: Although Dhaka is the divisional district like other divisions but initially Dhaka was taken as a different location because it is the capital of the Bangladesh, majority of people lives there so infection rate and death rate were exceptionally large than other divisions and districts. That is why Dhaka was so taken. However, as number of participants from this location was very small, therefore, during analysis participants from Dhaka were considered as participants from divisional district town and different tests were conducted accordingly.

Moreover, I made correction what you marked in manuscript draft.
