# Supplementary material for: Knowledge, attitude, practice, and fear level of Bangladeshi students toward Covid-19 after a year of the pandemic situation: A web-based cross-sectional study
Source: PLoS One. 2023 Feb 27;18(2):e0282282. doi: 10.1371/journal.pone.0282282 (PMC9970072; doi:10.1371/journal.pone.0282282)
Supplement: Attachment — Submitted filename: Response to Reviewers.docx [file pone.0282282.s007.docx]

Here I am writing our responses to each points raised by the reviewers.

Responses to Reviewer #1

 This was a well conducted study and the results were presented and interpreted in an appropriate manner. You should do some additional minor corrections. All the comments are provided in the revised manuscript draft. However, the authors need to extensively review and edit grammars and writing. Please use help from a native English speaker, or from someone who is an expert user of English.

Author: Thanks for your valuable comments. We have corrected all the marked issues as you suggested (Please vide the Revised manuscript with track changes). We (all authors) thoroughly revised the manuscript again and also taken help from a public health researcher expert in this field as well as expert user of English. If further correction needed, please inform us we will correct according to your suggestion.

Responses to Reviewer #2

Substantial language edits are still required.
Research ethics: It is not clear how they have taken assents from the participants of under-18. Guardians' consents are also required in this case. It is not clear how they have reached out the guardian of under-18 students while conducting a web-based survey. This needs to be explained in the manuscript.
The author mentioned that the study was approved by the ethical review committee of
Rajshahi University. The authors are suggested to provide the approval letter as a supplementary file for strengthening the credibility of ethical issue of the study.

Author: Thanks for your valuable suggestion. I have cleared how we had reached out the guardian of under 18 year old participants and other ethical issues in the manuscript [Please vide line (123-124) & (199-204)].
