# Supplementary material for: Knowledge, attitude, practice, and fear level of Bangladeshi students toward Covid-19 after a year of the pandemic situation: A web-based cross-sectional study
Source: PLoS One. 2023 Feb 27;18(2):e0282282. doi: 10.1371/journal.pone.0282282 (PMC9970072; doi:10.1371/journal.pone.0282282)
Supplement: Attachment — Submitted filename: Response to Reviewers.docx [file pone.0282282.s008.docx]

Dear editor,

Here I am writing our responses to each points raised by the reviewers.

Responses to the queries of Reviewer #1:

Thanks for your valuable comments. According to your suggestion, all the authors critically revised the manuscript again. Besides, we requested another expert (name mentioned on the Acknowledgment section) to revise our manuscript. Hopefully, his contribution would add significant value to this manuscript. (All the changes have been marked on the “Revised Manuscript with Track Changes”)

Responses to the queries of Reviewer #2:

Thanks for your valuable suggestion. I provided the approval letter (scanned copy of the original letter) as a supplementary file for strengthening the credibility of ethical issue of the study.
