# Supplementary material for: Knowledge, attitude, practice, and fear level of Bangladeshi students toward Covid-19 after a year of the pandemic situation: A web-based cross-sectional study
Source: PLoS One. 2023 Feb 27;18(2):e0282282. doi: 10.1371/journal.pone.0282282 (PMC9970072; doi:10.1371/journal.pone.0282282)
Supplement: Attachment — Submitted filename: Response to Reviewers.docx [file pone.0282282.s010.docx]

**Response to Reviewers:**

**Reviewer #1:** Despite repeated request the authors failed to improve the manuscript’s language and grammar. However, as the study have some interesting findings, I’ve extensively read and put my comments and corrections in the manuscript. Please incorporate them to improve the style.

**Author:** I am expressing my sincere gratitude for your valuable comments and corrections. I have incorporated all the corrections you had made to the manuscript. Please see the “Revised Manuscript with Track Changes”. Please let me know, if further correction is needed.

**Reviewer #2:** Editing in English is still required.

It is suggested that the authors seek assistance from someone who is proficient in written English.

**Author:** Thanks for your suggestion. Previously, I had taken assistance from two person who are proficient in written English but probably they could not perceive the demand of this journal. This time, I took assistance of them again and changed some part of the manuscript according to their suggestions and the suggestions made by “Reviewer #1”. Hopefully, this revised manuscript will meet the specifications of the journal. If it fails again, please let me know.
