# Supplementary material for: Knowledge, attitude, practice, and fear level of Bangladeshi students toward Covid-19 after a year of the pandemic situation: A web-based cross-sectional study
Source: PLoS One. 2023 Feb 27;18(2):e0282282. doi: 10.1371/journal.pone.0282282 (PMC9970072; doi:10.1371/journal.pone.0282282)
Supplement: Attachment — Submitted filename: Response to Reviewers.docx [file pone.0282282.s011.docx]

**Response to Reviewers:**

**Reviewer #1:** Thanks to the authors that they incorporated all of the comments in the previous review. However, the manuscript still needs improvement in its description and presentation (i.e., English). I suggest the authors to take help from a native English speaker or a professional English editing service.

**Author:** Thanks for your suggestion. To speak frankly, we are very new researchers and this is our very first manuscript that has come at this stage. We had no known native English speakers as well as no fund for taking any professional English editing service. We are mainly taking help from our teachers who are Phd holders and established researchers. Therefore, we request you kindly to consider our situation. If we could publish this manuscript, that would give us more confidence for future researches.

**Reviewer #2:** The language in this revised manuscript is still unclear, although the reviewers have repeatedly suggested the authors edit the language. Examples are numerous:

**Author:** According to your suggestion, we reviewed our manuscript by another expert. According to his suggestion, we made changes many sentences to remove ambiguity. Please, see the “Revised Manuscript with Track Changes”.

Lines (65-70): 'If this Delta variant starts to escalate vehemently in Bangladesh as it did in India and Nepal, people stay lackadaisical in maintaining public health hygiene, and the government continues to develop an ill-conceived plan of action and fails to ensure proper implementation of preventive measures, then, the number of cases detected per day could rise to an uncontrollable digit, which can throw the situation into turmoil (4).'

**Author:** I have made changes. Please, see the Lines (73-76).

Lines (81-85) 'A review of some previous studies conducted in Bangladesh reflected the truth that in last year (2020) Bangladeshi people were comparatively less knowledgeable, had a less positive attitude toward Covid-19, and as a consequence had less frequent preventive practice, which was consistent with a study of Pakistan but inconsistent with China and Saudi Arabia (7-14).'

**Author:** I have made changes. Please, see the lines (86-88).

Lines (130-134): ' A structured questionnaire was prepared in a Google Form and a link was generated, shared with all authors and other volunteers who were instructed properly as to how well they can use this form to recruit data with adequate consent from the participants and from the guardians of the participants aged under 18 years old.

**Author:** I have made changes. Please, see the lines (128-130).

Lines (263-266): 'Mann-Whitney and Kruskal-Wallis tests showed that knowledge scores do not vary with any socio-demographic variables included in our study, indicating all the groups of a variable
had quite similar knowledge on Covid-19 disease (Table 4).

**Author:** I have made changes. Please, see the lines (258-260).
