# Supplementary material for: Knowledge, attitude, practice, and fear level of Bangladeshi students toward Covid-19 after a year of the pandemic situation: A web-based cross-sectional study
Source: PLoS One. 2023 Feb 27;18(2):e0282282. doi: 10.1371/journal.pone.0282282 (PMC9970072; doi:10.1371/journal.pone.0282282)
Supplement: Attachment — Submitted filename: Response to Reviewers.docx [file pone.0282282.s012.docx]

**Additional Editor Comments:**

The discussion section should be revised accordingly.

The figure citation from discussion section should be removed.

**Author:** Due to some confusion, I have not removed figure citation from discussion section. I am not clear whether I should completely remove Fig. 3 and figure citation from the manuscript or replace in other section. I think, there should have a figure for clear conception. However, I will change according to your decision.

Sub headings from discussion should be removed. PLOS ONE manuscript writing guidelines should be followed. Can be found here, <https://journals.plos.org/plosone/s/submission-guidelines>

**Author:** According to suggestion, I removed all sub headings from discussion.

The authors should follow the reference style according to PLOS ONE guidelines.

**Author:** Reference style is ‘Vancouver’ which is in accordance with Plos One guidelines.
