# Supplementary material for: Knowledge, attitude, practice, and fear level of Bangladeshi students toward Covid-19 after a year of the pandemic situation: A web-based cross-sectional study
Source: PLoS One. 2023 Feb 27;18(2):e0282282. doi: 10.1371/journal.pone.0282282 (PMC9970072; doi:10.1371/journal.pone.0282282)
Supplement: Attachment — Submitted filename: Reviewer comments.docx [file pone.0282282.s004.docx]

Overall comment:

The topic is of interest but several papers on this issue have already been published recently, so it is uncertainty to what extent the article will contribute to add knowledge to this field. For example, please see the link below:

<https://journals.plos.org/plosone/article?id=10.1371/journal.pone.0239254>

Substantial English edits are required

Specific comments to the authors:

Title:

Better to omit the word ‘Present’ from the title. It is suggested to keep the title as ‘Knowledge, attitude, practice, and fear level of Bangladeshi people towards covid-19 after a year of the pandemic situation: a web-based cross-sectional study’

This study has been conducted mainly among the students (out of 402 samples, 382 are students) and does not necessarily represent Bangladeshi people as a whole, therefore, it is suggested to make changes in the title of the manuscript.

Abstract:

Age of the study participants ranged from 16-30 meaning that all were not adults, so the authors are requested to make correction in the abstract and report the percentage of the participants <18 years.

Please report the exact percentage, not like more than 90%.

Background:

Overall, the background section needs to be re-written. It does not well justify the study.

Please do not mention individual’s name that can be identified easily and give reference of the statement. if it is personal communication, follow the reference style for that as well (lines 59-65)

Please mention the exact year instead of ‘this year’ (line 76)

The authors should mention ‘The Government of Bangladesh (GoB)’ throughout the paper, instead of ‘Bangladesh’s government’

Please mention the exact year instead of ‘last year’ (line 82)

Please give reference for the statement (lines 81-86) and follow the reference style instructed in author guideline

I don’t see any figure or diagram in the document (missing!)

Methodology:

It is not clear how the participants were approached and selected.

It seems that the study was conducted among the Bangladeshi student. Please make it clear, make correction accordingly throughout the paper.

Please write ‘participants’ or ‘respondents’ instead of study ‘subjects’

Income is not sufficient to determine social status and classify as ‘lower class, middle class and high class’. It is suggested to keep it simple as ‘income’ and categorize the income using range.

Ethical consideration:

The authors should mention how they have taken assents from the participants of <18 years

Data management and analysis:

The author should mention why they performed different statistical test and what they found from each test (i.e., how was the distribution of data under the variable(s)?)

What was/were the outcome(s) of interest in regression model? what independent variables were taken into consideration? And level of significance considered during keeping the variables in final model.

The authors performed MLR; why didn’t they report adjusted OR?

Results:

Overall, the results are not well interpreted. The authors have been suggested organize, present and interpret the results in scientific manner. Examples are numerous:

Please mention the equivalent grades instead of ‘honors and masters’ (line 207)

It seems that the participants were the students, not from other occupations. The authors have been suggested to rewrite and interpret the result section accordingly.

‘Participants’ Overall correct answer rate was 89.6% [Mean Score (MS) ± Standard 218 Deviation (SD) = 10.75±1.20 out of 12] while the correct answer rate was 65% to 219 100%, indicating participants had appreciable knowledge on covid-19.’ Interpretation of the results is confusing. Please rephrase.
